# Telomere-related DNA damage response pathways in cancer therapy: prospective targets

**DOI:** 10.3389/fphar.2024.1379166

**Published:** 2024-06-07

**Authors:** Liting Gu, Mingdi Liu, Yuning Zhang, Honglan Zhou, Yishu Wang, Zhi-Xiang Xu

**Affiliations:** ^1^ Key Laboratory of Pathobiology, Ministry of Education, Jilin University, Changchun, Jilin, China; ^2^ Department of Urology, The First Hospital of Jilin University, Changchun, Jilin, China

**Keywords:** alternative lengthening of telomeres, cancer, DNA damage response, telomerase inhibitor, telomere

## Abstract

Maintaining the structural integrity of genomic chromosomal DNA is an essential role of cellular life and requires two important biological mechanisms: the DNA damage response (DDR) mechanism and telomere protection mechanism at chromosome ends. Because abnormalities in telomeres and cellular DDR regulation are strongly associated with human aging and cancer, there is a reciprocal regulation of telomeres and cellular DDR. Moreover, several drug treatments for DDR are currently available. This paper reviews the progress in research on the interaction between telomeres and cellular DNA damage repair pathways. The research on the crosstalk between telomere damage and DDR is important for improving the efficacy of tumor treatment. However, further studies are required to confirm this hypothesis.

## 1 Introduction

The maintenance of genomic integrity is a fundamental feature of cellular physiology (Maréchal and Zou, 2013). However, DNA damage occurs continuously in cells exposed to numerous extrinsic sources, including ionizing radiation, ultraviolet irradiation, and chemical exposure, as well as intrinsic sources, including metabolic responses, oxidative stress, and replication errors, that eventually result in single- or double-stranded DNA breaks (DSBs) (Basu et al., 2012; De Falco and De, 2021; Cheng et al., 2022). In normal cells, several DNA damage response (DDR) mechanism participate in maintaining cell viability and genomic stability. The primary types of DDR include homologous recombination (HR), base excision repair (BER), non-homologous end-joining (NHEJ), break-induced replication (BIR), mismatch repair (MMR), nucleotide excision repair (NER), direct repair (DR), and single-stranded annealing (SSA), which can facilitate pinpoint DNA restoration, identify DNA lesions, prevent cell division-related processes, and enhance aberrant apoptosis (Beard et al., 2019). Missing information in the DDR pathway can lead to mutations that result in genomic destabilization, thereby contributing to carcinoma formation.

DDR functions in two ways: by preventing cells from entering mitosis until repair is completed via activating DNA damage checkpoints and by coordinating and activating various repair pathways and inducing metabolic reprogramming. DNA-dependent protein kinase (DNA-PK) and capillary dilated ataxia mutated (ATM) and ATM and Rad3-related (ATR) kinases, which belong to the PI3K-related kinase (PIKK) family, play essential roles in this process. Among them, DNA-PK and ATM are primarily responsible for DSB repair, whereas ATR repairs the damage induced by DNA replication (Roos et al., 2016; Blackford and Jackson, 2017). Telomeres are nuclear protein complexes comprising TTAGGG repeats located at the ends of linear chromosomes. Under normal conditions, the telomere is covered with the shelterin protein complex, which comprises six proteins, including TRF1, TRF2, POT1, RAP1, TPP1, and TIN2 (de Lange, 2005). Their essential function is to protect chromosomal ends from recognition by free ends generated by DSB, which incorrectly activate DDR mechanisms that result in DNA degradation, end-to-end fusion, and genomic instability (Li et al., 2022). Therefore, telomeres are strongly associated with DDR.

Shelterin protein complexes are involved in distinguishing telomere ends from damaged DNA and can induce DDR. The TRF2 and POT1 proteins suppress ATM- and ATR-mediated DDR pathways, respectively, thereby avoiding the onset of the response. As cells divide, telomeres become progressively shorter, and when they reach a certain level of shortening, the ATM- and ATR-mediated DDR pathways are activated, which results in cell death or senescence. However, several studies have shown that proteins that are associated with DDR appear at telomeres and are directly or indirectly involved in telomere maintenance. Moreover, defects in DSB repair proteins, such as ATM, Ku, DNA-PKcs, RAD51, and MRN complexes, lead to the mismetabolism of telomeres. Gabriel Arantes Dos Santos et al. showed that upregulation of shelterin and CST (Cdc13/Ctc1, Stn1, Ten1) led to telomere lengthening and promoted invasion of prostate cancer cells (Dos Santos et al., 2024). Importantly, shelterin links to tumor immunity and predicts response to PD-1 blockade immune therapy (Luo et al., 2021). Therefore, functional telomeres interact with DDR proteins (Slijepcevic, 2006).

The activation of telomere maintenance mechanisms, including telomerase and the alternative lengthening of telomeres (ALTs), is essential for tumor cell growth, although its regulatory mechanisms are not completely understood (Kaul et al., 2021). ALT is a BIR-based mechanism that elongates telomeres in a subset of human cancer cells (Silva et al., 2021). Telomeres in ALT + cells are inherently unstable and prone to replicative stress and spontaneous DSB formation, which causes the repeated activation of DDR (Feretzaki et al., 2020). In ALT + cancer cells, telomeric repeats that contain long noncoding RNA (TERRA) interact with DNA repair proteins involved in several DNA repair pathways, including NER, DSB, and BER, indicating a strong link between DDR and telomere function (Guh et al., 2022). Therefore, telomeres in normal cells need to avoid DDR employment. In contrast, telomere replication and protection require the involvement of DDR-related proteins (Verdun and Karlseder, 2007). Thus, this review illuminates the relationship between telomeres and cellular DDR and provides clues on how to target telomere-associated DDR for cancer treatment.

## 2 Multiple DDRs and telomere-related DNA repairing

DNA damage, in the form of DNA base abnormalities or DSBs, may produce mutations in cells, promote malignant proliferation, and induce cancer. However, such mutations can be avoided if a precise DNA repair system can recognize and repair the damage before replication. In particular, DDR disruption occurs during cancer progression and can be used as a target for cancer treatment. Functional abnormalities in various proteins responsible for repair pathways can also contribute to the build-up of damage and, consequently, to the induction of cancer. Therefore, DNA repair is a key protective mechanism against malignant cell proliferation and cancer development (Nagel et al., 2017; Zimmermann et al., 2022).

Four primary types of DDR are present in eukaryotes: NER, BER, MMR, and double-strand break repair (DSBR). NER excises large segments of damaged DNA, BER repairs damage to individual bases, MMR is used to repair base mismatches, and DSBR includes two mechanisms (i.e., HR and NHEJ). NHEJ directly attaches to the fractured ends without a template, whereas HR requires complete sister chromatids as a repair pattern. DR is another DDR system that can repair certain forms of base damage without removing the bases (Maréchal and Zou, 2013; Caldecott, 2020; Bayley et al., 2022). BIR relies on homologous sequence templates for DNA synthesis and repair, particularly for repairing one-ended DSBs. SSA is a repair process that occurs when homologous sequences are present in the same direction at both ends of a DSB.

### 2.1 HR

HR is a multistep process that prevents genomic instability and maintains cellular homeostasis. After DSBs occur, BRCA1 promotes HR pathways. First, the MRN complex (mre11-rad50-nbs1) activates this pathway by coupling to DSB (Belan et al., 2022). The MRN complex acts synergistically with BRCA1 and CtIP endonucleases to mediate DNA end resection (Kishkevich et al., 2022). Furthermore, the MRN complex triggers ATM, which in turn initiates PALB2, BRCA1, and BRCA2 expressions (Murciano-Goroff et al., 2022; Peng et al., 2023). Subsequently, RAD51 loads onto the DNA damage site to form a nucleoprotein, which further invades sister chromosomes to search for orthologous DNA sequences that can be used as templates for novel DNA synthesis (Cleary et al., 2020; Murciano-Goroff et al., 2022).

By losing ATM in cancer cells, HR is undermined; therefore, when DNA is damaged, these cancer cells depend on the rest of the DDR pathway for repair (Kaminski et al., 2022). Cancers with specific HR defects can be treated with targeted agents that inhibit HR proteins.

In cancer cells, a mechanism of telomere lengthening via homologous targeted repair (i.e., ALT) is similar to HR and triggers DNA repair to maintain telomere length (Kaminski et al., 2022). TERRA is a transcription factor involved in telomere elongation. Chia et al. reported that several HR-related proteins, including RAD50, BRCA1, WRN, ATR, and WRNIP1, are potential TERRA-interacting proteins (Guh et al., 2022). TERRA initiates RAD51-dependent strand invasion (Feretzaki et al., 2020), whereas BRCA1 binds to and represses TERRA transcription (Vohhodina et al., 2021). A strong correlation exists between telomere function and the DNA damage response, particularly HR, which might be a potential therapeutic target for ALT cancer treatment.

### 2.2 NHEJ

NHEJ is a DDR pathway for the repair of DSBs and is activated by 53BP1, RIF1, and the shieldin complex. NHEJ includes classical non-homologous end-joining (cNHEJ), alternative non-homologous end-joining (alt-EJ), and MMEJ (Gómez-Cabello et al., 2022). cNHEJ utilizes nonspecific ligation to correct DNA breaks, resulting in error-prone repair that can occur at any time in the cell cycle (van de Kooij et al., 2022). cNHEJ is initiated via binding of the Ku70–Ku80 (also known as XRCC6–XRCC5) heterodimer to DSB ends (Luedeman et al., 2022). This event activates DNA-PK, which, in turn, activates a multi-protein complex of XRCC4, Artemis, and DNA ligase. Although the NHEJ mechanism is simpler than the HR mechanism, it can sometimes result in rearrangements, whereas the HR mechanism does not produce errors (Findlay et al., 2018). Alt-EJ primarily utilizes microhomologous fragments (2–25 bp) of the damaged region to generate an annealing reaction and remove nonhomologous ssDNA (Oanh et al., 2022). Alt-EJ is poly ADP-ribose polymerase 1 (PARP1)-dependent, and polymerase θ (Polθ) mediates break repair after MRN complex and PARP1-binding double-strand breaks. When HR is defective in tumors, it causes an enhanced reliance on alt-EJ. The correlation between the effect of alt-EJ on Polθ indicates that Polθ inhibitors are likely to be potent in HR-deficient tumors (Findlay et al., 2018; Scully et al., 2019; Shibata and Jeggo, 2020; Murciano-Goroff et al., 2022).

Ribes-Zamora et al. have determined the role of shelterin in suppressing the NHEJ function of Ku in human telomeres (Ribes-Zamora et al., 2013). Telomere fusion occurs when NHEJ acts on uncapped telomeres (Ueno, 2023). As the center of telomere maintenance and structure, the complex between TRF2 and Rap1 blocks NHEJ, and together with DNA-PK, inhibits telomere end-joining (Arat and Griffith, 2012). NHP2 is a component of the telomerase–holoenzyme complex. In telomere RNA subunit (hTR)-expressing ALT + cells, NHP2 is downregulated, and 53BP1 foci at telomeres are increased. The depletion of NHP2 in hTR-expressing cells rarely reduces the total 53BP1 level, but does decrease TIF reduction compared to NHP2 depletion in non-hTR-expressing cells (Raghunandan et al., 2021). Therefore, NHEJ factors are attractive targets for cancer therapies because of the reliance of tumor cell division on DNA repair mechanisms.

### 2.3 BIR

BIR primarily repairs single-ended DSBs similar to those resulting from telomere encroachment or replication fork crashes (Kockler et al., 2021). BIR was first reported in bacteriophage T4 and occurs in both mammalian cells and humans (Kreuzer, 2000). One study found that the overexpression of oncogenes activates BIR in human cells, resulting in chromosomal rearrangements (Elango et al., 2019). In ALT + cells, BIR is active during the G2 stage of the cell cycle and RAD52 is recruited to the replication stress site, a process that requires the two regulatory subunits of DNA polymerase δ, namely, POLD3 and POLD4 (Silva et al., 2019). ALT is a BIR that functions through both RAD52-and non-dependent processes. (Verma et al., 2019). In ALT cells, RAD52 is primarily responsible for D-loop formation and mediates the RAD52-dependent BIR process, whereas RAD51AP1 is primarily responsible for TERRA-mediated R-loop formation in telomeres, which promotes G4 formation. Subsequently, G4 promotes R-loop-to-D-loop conversion, which promotes ALT (Kaminski et al., 2022; Yadav et al., 2022). Moreover, BIR can trigger the SUMOylation of PIAS4-mediated TRF2, and the deprivation of PIAS4 renders APB devoid of repair proteins, which in turn compromises the synthesis of ALT telomeres (Zhang et al., 2021). Therefore, understanding the role of BIR in the treatment of ALT cancer cells is vital (Elango et al., 2017).

### 2.4 SSA

SSA is a double-stranded oligonucleotide with a 3′ overhang of three random nucleotides that can be efficiently ligated to the 3’ end of single-stranded DNA using T4 DNA ligase (Wu et al., 2018). Similar to the alt-EJ mechanism, SSA requires homologous DNA sites to catalyze DSB repair. However, SSA can occur over long stretches of DNA and result in large deletions that can cause intrachromosomal translocations. Mechanistically, SSA is inhibited by RAD51. Unlike alt-EJ, which requires PARP and Polθ, SSA requires RAD52 to influence the annealing of the homologous stretches of ssDNA (Scully et al., 2019; Vancevska et al., 2020; Subecz et al., 2021).

The mammalian ERCC1/XPF endonuclease plays an important role in DSB repair via SSA (Kim et al., 2020). TERRA triggers XPF localization to telomeres and results in FANCM deficiency, eventually leading to DSBs (Guh et al., 2022). SLX4, a coordinator of multiple DNA structure-specific endonucleases, plays important roles in several DNA repair pathways. The Slx4-Rad1 complex is required for the SSA pathway, in which the Mec1/Tel1-dependent phosphorylation of Slx4 is essential (Saito et al., 2009). SLX4 cooperates with XPF for interstrand DNA crosslink repair and is required for XPF-mediated DDR at ALT telomeres (Guh et al., 2022). Therefore, therapies targeting the SSA repair pathway may be useful for treating ALT cancer cells (Pfitzer et al., 2019).

### 2.5 BER and NER

BER is initiated by an impaired base and is substituted by de novo-synthesized DNA. Then, APE (depurine/depyrimidine nuclease) cleaves it to form a 3′OH end at the site of damage (Szymanski et al., 2022; Tang et al., 2022). Finally, DNA ligase and polymerase are used to bridge these gaps (Farag et al., 2022). The NER mechanism involves the removal of damaged DNA by the excision repair cross-complementary protein 1 (ERCCI), which is substituted with normal DNA replication (Elango et al., 2017; Scully et al., 2019). Defects in BER are correlated with premature aging, and BER genes are overexpressed in various cancers, such as POLβ, XRCC1, and APE1, thus indicating that BER is essential for genome maintenance (Somuncu et al., 2020).

XPF is an NER factor with nucleic acid endonuclease activity and is the most enriched TERRA-binding protein according to mass spectrum results (Guh et al., 2022). XPF can generate DSBs while promoting DDR in ALT telomeres (Guh et al., 2022). Yang et al. discovered that the principal mechanism of telomere replication may be linked to TFIIH, because as an NER element, TFIIH is an important factor in TRF1 and its absence results in several telomere replication phenotypes (Yang et al., 2022).

### 2.6 MMR

MMR restores the nucleotide sequence in DNA molecules with mismatched bases. When microsatellite instability (MSI) occurs in an organism, MMR proteins repair the errors. Normal functioning of the MMR protein repairs the MSI and maintains microsatellite stability; however, when the MMR protein is absent, MSI is not repaired, and it will gradually accumulate, thus resulting in high MSI. MSI is typically found in colorectal cancer but can also occur in gastric and endometrial cancers (Scully et al., 2019; Shibata and Jeggo, 2020; Ngo et al., 2021).

Recent literature suggests that the loss of MMR function may play a significant role in ALT activity in human cancer cell lines; however, this correlation has not been confirmed in human primary tumors. Furthermore, the loss of MMR function is associated with ALT, improved organism survival, and health in yeast and mice, thus supporting the role of the loss of the MMR pathway in promoting the development of ALT (Stundon et al., 2022). MMR is initiated by one of two heterodimers: MSH2/MSH6 (MutSα) and MSH2/MSH3 (MutSβ). MutSα binds to base–base mismatches or the insertion and deletion loops of 1–3 nt, whereas MutSβ binds to insertion and deletion loops containing up to 16 nt (Kunkel and Erie, 2015). A recent study revealed that MutSα restricts telomere extension via ALT-associated homology-directed repair in human cancer cells (Barroso-González et al., 2021). MutSβ precludes the aggregation of R-loops and telomeric G-quadruplex (G4) structures (Sakellariou et al., 2022). Additionally, SLX4 is associated with the proteins MSH2-MSH1 and TRF2 (Ouyang et al., 2015). These associations suggest a link between telomeres and MMR, thus offering potential therapeutic alternatives for cancer treatment.

## 3 Protective role of the shelterin complex in DDR

The shelterin complex at the telomere ends forms a protective T-loop, which alters the end of the chromosome similar to that of the recombinant D-loop, thus concealing the 3′one-stranded DNA overhanging ends and preventing the false activation of DDR. Most somatic cells have progressively shorter telomeres, but carcinomas can sustain telomere length by upregulating telomerase activity or using the ALT mechanism (Barnes et al., 2023). How does DDR at telomere ends in tumors protect cells from overproliferation and promote tumorigenesis? Shelterin inhibits multiple DDR pathways, and different shelterin subunits are involved in diverse reparative pathways and signaling. For instance, the absence of TRF2 triggers ATM signaling, which leads to telomere fusion. In contrast, in the absence of POT1, ATR signaling is activated at telomeres, but the ATM signaling pathway remains inhibited. HR inhibition results in telomeric sister chromatid exchange, which involves the presence of concurrent RAP1 and POT1 proteins in the telomere. TRF2 and another shelterin protein also inhibit the ALT–NHEJ pathway (Doksani and de Lange, 2014; Doksani and de Lange, 2016). The CST complex in mammals has been reported to boost telomere replication but has no direct role in inhibiting telomere DDR (Gu and Chang, 2013). Disruption of the DDR can promote cancer development and progression; thus, the destruction of DDR pathways in cancer cells could be used to treat cancer.

## 4 Damaged DNA-targeted therapies in cancer

### 4.1 Relevant treatments of telomere and drugs available

#### 4.1.1 PARP inhibitors

The development of PARP inhibitors has resulted in synthetic lethality. The binding of PARP1 to single-stranded DNA breaks produced during BER forms the basis of a synthetic lethal interaction with HR defects (de Vos et al., 2012). Preclinical and clinical studies on PARP inhibitors have revealed additional mechanisms of their activity (Xue et al., 2022). PARP inhibitors trap the PARP enzyme at damaged DNA sites, thereby influencing the prevention of essential cellular processes, including DNA repair and transcription. However, in HR-deficient cells, the trapped PARP–DNA complex is lethal (Illuzzi et al., 2022). In HR-deficient cell lines, PARP inhibitors are currently available, including niraparib (Chi et al., 2023), talazoparib (Agarwal et al., 2023), rucaparib (Fizazi et al., 2023), olaparib (Robson et al., 2017), veliparib (Coleman et al., 2019), AZD5305 (78) and IMP4297(79). Clinical trials of these inhibitors have been approved and used for several cancers, including ovarian, breast, and pancreatic cancers. However, the acquired resistance to PARP inhibitors in clinical settings remains to be resolved (Vancevska et al., 2020; Vernì, 2022) (Table 1).

**TABLE 1:** List of DDR inhibitors in clinical trials study.

Inhibitor	Drugs	Phase	Target	References
PARP inhibitors	Niraparib	Ⅱ, Ⅲ	OC, BC, prostate cancer	Agarwal et al. (2023)
	Olaparib	Ⅲ	BC, OC, mCRPC, pancreatic cancer, TNBC	Robson et al. (2017)
	Talazoparib	Ⅱ, Ⅲ	BC, mCRPC	Agarwal et al. (2023)
	Rucaparib	Ⅱ, Ⅲ	OC, mCRPC	Agarwal et al. (2023)
	Veloparib	Ⅲ	NSCLC	Coleman et al. (2019)
	AZD5305	Ⅰ, Ⅱ	solid tumors	Zheng et al. (2022)
	IMP4297	Ⅰ	SCLC	Hu et al. (2023)
ATR inhibitors	Ceralasertib (AZD6738)	Ⅰ	HNSCC	Vendetti et al. (2018)
		Ⅱ	OC, solid tumors, SCLC	Biegała et al. (2023)
		Ⅲ	NSCLC	Vendetti et al. (2018)
	Berzosertib (VX970, M6620)	Ⅰ	OC, solid tumors	Telli et al. (2022)
		Ⅱ	SCLC	Takahashi et al. (2023)
	Elimusertib (BAY1895344)	Ⅰ	solid tumors	Harold et al. (2023)
	Tuvusertib (M1774)	Ⅰ	solid tumors	Yap et al. (2024)
	Camonsertib (RP-3500)	Ⅰ, Ⅱ	solid tumors	Yap et al. (2023)
APE1 inhibitors	Methoxyamine (TRC-102)	Ⅰ, Ⅱ	solid tumors	Eads et al. (2021)
	E3330 (APX3330)	Ⅰ	solid tumors	Fishel et al. (2019)
	Lucanthone	Ⅱ	glioblastoma	Radin et al. (2024)
DNA-PK inhibitors	AZD7648	Ⅰ, Ⅱ	solid tumors	Radin et al. (2024)
	LY3023414 (samotolisib)	Ⅱ	solid tumors, NSCLC, TNBC, prostate cance, PDAC	Sweeney et al. (2022)
	Nedisertib (peposertib)	Ⅰ, Ⅱ	SCLC, rectal cancer	Samuels et al. (2024)
	Voxtalisib (XL765, SAR245409)	Ⅰ	NSCLC, glioblastoma	Wen et al. (2015)
		Ⅰ, Ⅱ	BC	Blackwell et al. (2015)
		Ⅱ	OC, lymphoma	Brown et al. (2018)
CHK1/CHK2 inhibitors	LY2603618 (rabusertib)	Ⅰ	HNSCC	van Harten et al. (2019)
	LY2880070	Ⅰ	PDAC	Huffman et al. (2023)
	Prexasertib (LY2606368)	Ⅱ	OC, SCLC	Konstantinopoulos et al. (2022)
	PHI-101	Ⅰ	OC	Park et al. (2022)
	GDC-0425	Ⅰ	solid tumors	Infante et al. (2017)
WEE1 inhibitors	AZD1775 (adavosertib)	Ⅱ	OC, SCLC, NSCLC, pancreatic cancer, TNBC	Fu et al. (2023)
	ZN-c3	Ⅰ, Ⅱ	pancreatic cancer, OC	Schutte et al. (2023)
		Ⅱ	AML	Huang et al. (2021)

Abbreviations: PARP, poly (ADP-ribose) polymerase; OC, ovarian cancer; BC, breast cancer; mCRPC, metastatic castrationresistant prostate cancer; NSCLC, non-small cell lung cancer; SCLC, small cell lung cancer; ATR, ataxia telangiectasia and Rad3 related protein; HNSCC, head and neck squamous cell carcinoma; AML, acute myeloid leukemia; TNBC, triple negative breast cancer; DNA-PK, DNA-dependent protein kinase; PDAC, pancreatic ductal adenocarcinoma.

PARP inhibitors have been reported to cause TRF2 to decapitate telomeres, resulting in the stimulation of incorrect NHEJ repair in ALT-positive cancer cells. Loss-of-function ATRX and/or DAXX mutations have been found in ALT-positive cancer cells. Currently, no drugs related to ATRX are being investigated in clinical trials (Cavalcante et al., 2021; Qin et al., 2022).

#### 4.1.2 Targeting ATM and ATR

As an apical DDR kinase, ATM regulates DSB repair in various cell types. Mechanistically, single-ended DSBs caused by PARP and topoisomerase one inhibitors require HR for accurate repair. The deletion of ATM signaling causes delayed end resection and repairs single-ended DSBs via NHEJ, resulting in irregular chromosome fusion and tumor cell death. ATM is considered a tumor suppressor and may lead to *de novo* tumor formation in various tissues when exposed to ATM inhibitors for prolonged periods (Pobiega et al., 2021). ATM defects or mutations are commonly found in solid tumors and B-cell lymphomas. Therefore, ensuring that the therapeutic benefits of ATM inhibition outweigh the therapeutic risks are important (Mukherjee et al., 2018; Scully et al., 2019; Subecz et al., 2021). A Phase I trial has already evaluated the ATM inhibitor AZD0156 as both a monotherapy and in combination with the PARP inhibitors irinotecan and olaparib, which is another cytotoxic agent (Davis et al., 2022; Qin et al., 2022; Wong et al., 2022).

During the S phase, ATR ensures accurate DNA replication by regulating the initiation of ignition and fork progression. ATR inhibitors can also increase replication fork arrest, induce chromosomal breakage, and cause cytotoxicity (Ouyang et al., 2015). The sensitivity of cancer cells to ATR inhibitors, which is caused by the overexpression of the oncogenic protein E1, is higher than that of other cell lines. Therefore, ATR inhibitors are often used to treat PARP inhibitor-resistant tumors (Kim et al., 2022). Cancer cells with BRCA1 mutations can overcome the toxicity of PARP inhibitors by loading DSB with BRCA1-independent RAD51, thereby overcoming drug resistance. ATR inhibitors block BRCA1-independent function and re-sensitize tumor cells to PARP inhibition *in vitro*. Preclinical data showed that berzosertib (also known as M6620 and VX-970) was the first ATR inhibitor to reveal that lung cancer cells were primarily sensitive to chemotherapeutic agents. The drug can result in the collapse of replication forks, such as cisplatin and gemcitabine (*in vitro*), and improve antitumor activity when combined with cisplatin (*in vivo*) (Perkhofer et al., 2021; Scheper et al., 2022; Telli et al., 2022; Viol et al., 2022; Takahashi et al., 2023). Other ATR inhibitors currently in clinical trials are as follows, for example, ceralasertib (AZD6738) (Vendetti et al., 2018; Biegała et al., 2023), elimusertib (BAY1895344) (Harold et al., 2023), tuvusertib (M1774) (Yap et al., 2024) and camonsertib (RP-3500) (Yap et al., 2023).

Replication stress occurs when damaged DNA impedes the progression of replication forks, leading to stagnation. If unrepaired, the stalled fork may deteriorate into a DSB, eventually facilitating the recruitment of DNA repair factors and engagement of HR mechanisms to lengthen telomeres. Therefore, ATM and ATR inhibitors are also used in cancer therapy to treat patients with ALT-positive cancers. The ATM inhibitor AZD0156 has shown selective toxicity in melanoma cells, neuroblastoma, and preclinical models of colorectal cancer (Davis et al., 2022; Qin et al., 2022; Wong et al., 2022; Yilmaz et al., 2023).

In contrast, other studies showed that ATM activation balances senescence, apoptosis, and autophagy. The G-quadruplex ligand 20A elicits global DNA damage and activates the ATM pathway in both cancer cells (HeLa) and xenograft mouse models (Beauvarlet et al., 2019). Other DDR-related inhibitors currently in clinical trials such as APE1 inhibitors (Fishel et al., 2019; Eads et al., 2021; Radin et al., 2024), DNA-PK inhibitors (Blackwell et al., 2015; Wen et al., 2015; Brown et al., 2018; Sweeney et al., 2022; Selvaraj et al., 2023; Samuels et al., 2024), CHK1/CHK2 inhibitors (Infante et al., 2017; van Harten et al., 2019; Konstantinopoulos et al., 2022; Park et al., 2022; Huffman et al., 2023), and WEE1 inhibitors (Huang et al., 2021; Fu et al., 2023; Schutte et al., 2023) are summarized in Table 1.

#### 4.1.3 Immune related telomere targeted therapy strategies

Ilgen Mender et al. elucidated for the first time the mechanism and potential clinical translational value of 6-thio-dG, a nucleoside analogue targeting telomere damage, in activating host DNA-cyclic GMP- AMP synthase (cGAS)–stimulator of interferon genes (STING) pathway -dependent immune cells to inhibit tumor growth (Mender et al., 2020). 6-thio-2′-deoxyguanosine (6-thio-dG) is a telomerase substrate precursor nucleoside analog that has been validated as a telomere-targeting strategy. Mechanistically, 6-thio-dG induces persistent telomere dysfunction and sequentially activates the ATR pathway, followed by ATM activation in telomerase-positive cells (Sengupta et al., 2018). Notably, ATR activation decreases after activation with 6-thio-dG, indicating the dual effect of DDR on telomere-related cell death. Previous studies have shown that treatment with low-dose THIO followed by anti-PD-1/PD-L1 immunotherapy eliminated advanced tumors in a clinical precursor cell model and generated cancer cell-specific immune memory, thus enabling the immune system to retain its activity against cancer cells after treatment cessation (Kodym et al., 2009). 6-thio-dG induces a high antitumor activity in chemotherapy-resistant tumor cells and mouse models (Mender et al., 2018). This co-treatment strategy is expected to provide clinical benefits to patients with small cell lung and colorectal cancers, as well as hepatocellular carcinoma, who have had unsuccessful first-line therapies (George et al., 2020; Yu et al., 2021). Due to structural similarities, many analogues previously used to inhibit human immunodeficiency virus (HIV) reverse transcriptase have also been found to inhibit the hTERT catalytic site. Drugs currently being investigated in telomerase-positive cancers include azidothymidine (AZT) and 5-methylcarboxy-indolyl-2′-deoxyribonucleoside 5′- triphosphate (5- MeCITP) (Gomez et al., 2012; Hernandez-Sanchez et al., 2019). GV1001 is a peptide derived from the reverse transcriptase subunit of telomerase (hTERT) that has been developed as a vaccine against a variety of cancers. GV1001 interacts with heat shock proteins (HSPs) and penetrates cell membranes to localize in the cytoplasm (Kim et al., 2016). It has been shown in the literature that chemotherapeutic agents combined with the GV1001 vaccine enhance the immune response but do not improve the overall survival of pancreatic cancer patients (Middleton et al., 2014). The UV1 vaccine consists of three synthetic long peptides and is a peptide vaccine against telomerase (Gerada et al., 2020). The UV1 vaccine has been tested in prostate cancer (Lilleby et al., 2017)、lung cancer (Brunsvig et al., 2020)and malignant melanoma (Aamdal et al., 2021), either alone or in combination with checkpoint inhibitors. A phase II trial of the UV1 telomerase vaccine in combination with ibritumomab and nifedumab together in pleural mesothelioma is currently underway, and the results have shown that the addition of the vaccine is more effective (Haakensen et al., 2024). Vx-001 is the first antitumor vaccine based on optimized cryptic peptides, targeting tumor antigen TERT, and its functional peptide is hidden inside the protein (Vassilis et al., 2013). Phase I/II trials of Vx-001 in patients with non-small cell lung cancer, melanoma, breast cancer, and many other cancers have been completed (Athanasios et al., 2014). In clinical trials, this vaccine demonstrated high hTERT-specific immune responses, good anti-tumor efficacy, good tolerability and few side effects (Menez-Jamet et al., 2016). INVAC-1 is a DNA plasmid encoding a modified hTERT protein for patients with relapsed or refractory solid tumors (Calvet et al., 2014). Phase I clinical trials of INVAC1 found that the vaccine was well tolerated, triggered hTERT-specific CD4^+^ and CD8^+^ T-cell responses, and blocked cancer progression in the majority of patients with relapsed or refractory solid tumors (Teixeira et al., 2020).

### 4.2 Other applications of telomere-related treatment in cancer

Owing to the prevalence of telomerase-positive cancer in all cancer patients, targeted telomerase therapy is considered a potential approach for cancer treatment. Several promising candidates are currently being investigated in clinical trials and pre-clinical studies (Table 2).

**TABLE 2:** Major telomere-targeting agents in preclinical and clinical development.

Drug targets	Drug	Study stage	Cancer types	Advantages	Disadvantages	References
Telomerase related telomere maintenance	GRN163L (imetelstat)	Phase Ⅰ, Ⅱ clinical trails	solid tumor, NSCLC, BC, pancreatic cancer, myelofibrosis, pediatric brain tumor, prostate cancer	Clinical efficacy in myelofibrosis and low-risk myelodysplastic syndromes	The effect on progression of other types of cancer is unclear and has serious side effects	(Fischer-Mertens et al., 2022; Djojosubroto et al., 2005)
	BIBR1532	Phase Ⅲ clinical trails	OC, NSCLC, BC, ATC, leukaemia, fibrosarcomas, endometrial cancer	Effectively inhibit tumors	Limited efficiency of sustained action	(Qin and Guo, 2022; Al-Karmalawy et al., 2023)
	6- thio- dG (THIO)	Phase Ⅱ clinical trails	SCLC, NSCLC, gliomas	Cross the blood–brain barrier	Elicit more rapid cytotoxicity	George et al. (2020), Yu et al. (2021)
	AZT	FDA approved	leukaemia, Kaposi sarcoma, lymphoma	FDA approved for the treatment of HIV	IC50 is high in non- virally induced cancer types	Gomez et al. (2012)
	5- MeCITP	Preclinical	lung cancer, colon cancer, pancreatic cancer, osteosarcoma	Fewer off- target effects, less toxic than AZT	Limited efficiency of sustained action	Hernandez-Sanchez et al. (2019)
	MST-312	Preclinical	BC, lung cancer, colon cancer, multiple myeloma	Exert anti- oncogenic effects *in vivo*	Low potency and slow onset of cytotoxic effects	Wu et al. (2020)
	NU-1	Preclinical	BC	Enhance the effects of radiation	Low potency and slow onset of cytotoxicity	Wu et al. (2020)
	GV1001	Phase Ⅲ clinical trails	PDAC, NSCLC, melanoma	Significantly prolonged survival in patients with CD8^+^ T-cell responses	Poor vaccine response rates	Middleton et al. (2014)
	UV1	Phase Ⅱ clinical trails	melanoma, NSCLC, prostate cancer	Improving the cancer killing effectiveness		(Lilleby et al., 2017; Brunsvig et al., 2020; Aamdal et al., 2021)
	Vx-001	Phase Ⅱ clinical trails	NSCLC	Long- lasting immune responses	Low response rates	Athanasios et al. (2014)
	INVAC1	Phase Ⅱ clinical trails	solid tumor	Safe, well tolerated		Teixeira et al. (2020)
	Telomestatin	Phase Ⅰ, Ⅱ clinical trails	multiple myeloma, neuroblastoma	Low toxicity	Poor solubility and chemical stability	Teng et al. (2021)
	TMPyP4	Preclinical	NSCLC	Effectively inhibits tumor growth	Affects the entire genome, including promoter regions of oncogenes	Iida et al. (2022)
	RHPS4	Preclinical	BC, glioblastoma	Effectively inhibit tumors	Promotes recombination and boosts ALT activity	Alessandrini et al. (2022)
	pyridostatin	Phase Ⅰ, Ⅱ clinical trails	BC, thyroid cancer, prostate cancer	Enhancing the anticancer activity of drugs that target DNA or inhibit its repair	Replication stress promotes recombination and drives ALT activity	Liu et al. (2022b)
Alternative lengthening of telomere	ATM inhibitors	Phase Ⅰ clinical trails	neuroblastomas	Selective toxicity	Limited effects which need to be combined with other drugs	Koneru et al. (2021)
	PARP inhibitors	Phase Ⅱ, Ⅲ clinical trails	BC, OC, pancreatic and metastatic prostate cancer	Effectively inhibits tumor growth	Highly toxic	Xue et al. (2022)
	ATR inhibitors	Phase Ⅰ, Ⅱ clinical trails	soild tumor, BC, OC, lung cancer	Effectively inhibits tumor growth	Highly toxic	Ouyang et al. (2015)
	PIP-199	Preclinical	osteosarcoma	The only reported small-molecule inhibitor of the FANCM–BTR	Selective toxicity	Lu et al. (2019)
	Tetra-Pt (bpy)	Preclinical	neuroblastoma	Effectively inhibit tumors		Zheng et al. (2017)

Abbreviations: 5- MeCITP, 5- methylcarboxyl-indolyl-2′- deoxyriboside 5′- triphosphate; AZT, azidothymidine; ATC, anaplastic thyroid cancer; OC, ovarian cancer; BC, breast cancer; NSCLC, non-small cell lung cancer; IC50, the inhibitory concentration 50; PDAC, pancreatic ductal adenocarcinoma.

Small-molecule telomerase reverse transcriptase (TERT) has been well studied and has achieved good practical prospects. BIBR1532 is a representative drug of this type that binds to TERT at the non-catalytic site and inhibits telomerase activity in a non-competitive manner (Liu B. et al., 2022). Its cytotoxicity is primarily caused by direct damage to the telomere structure, resulting in the loss of TRF2 binding, which induces telomere dysfunction, acts as a telomere end-to-end fusion, and increases p53 activation. The preclinical studies have shown that BIBR1532 is effective against several cancer cell lines, including breast cancer, fibrosarcoma, endometrial cancer, and leukemia (Qin and Guo, 2022; Al-Karmalawy et al., 2023). Due to the relative instability of epigallocatechin gallate (EGCG), improved TERT inhibitors synthesized from EGCG-related fractions have been developed (Wu et al., 2020). Among these, MST-312 has been confirmed to be involved in various types of cancers. In breast, lung, and colon cancers, MST-312 treatment significantly downregulates TERT expression, reduces telomerase activity, and results in telomere shortening. It can also lead to cell cycle arrest and apoptosis in cancer cells (Wu et al., 2020). However, MST-312 works only on cancer cells with short telomeres, according to the time required for telomere shortening to a critical length (90 d) (Fernandes et al., 2022). Nu-1 and erythromycin antibiotics are other TERT inhibitors that can directly bind to the TERT catalytic domain and block TERT transcription. However, these drugs have not been well studied and have only been investigated in early clinical research because of their low potency and slow onset of cytotoxicity (Ameri et al., 2019; Wu et al., 2020). G4 ligands inhibit telomerase binding to telomeric DNA, eventually inhibiting telomerase activity (Tiek et al., 2022). G4-stabilizing ligands include telomeric repressors, TMPyP4, RHPS4, pyridostatin and telomestatin (Teng et al., 2021; Alessandrini et al., 2022; Liu LY. et al., 2022; Iida et al., 2022). Owing to the high levels of G-rich DNA throughout the genome, particularly in the promoter regions of oncogenes, G4 ligands pose numerous risks when used as telomerase inhibitors and might have a significant off-target effect (Yan et al., 2021; Shankar et al., 2022; Tsai et al., 2022). Oligonucleotides can form stable double-stranded bodies with complementary DNA, thereby disrupting hTR function. Binding to the hTR sequence template effectively inhibits the catalytic action of telomeric repeat addition, thereby inhibiting telomerase activity. The representative drug in this category is imetelstat (GRN163L), which has been tested in different tumor models and is currently the only anti-telomerase oligonucleotide in clinical use (Djojosubroto et al., 2005; Fischer-Mertens et al., 2022).

Regarding ALT-positive cancer cells, the development of cancer drugs targeting ALT can be traced back to its upstream and downstream pathways, particularly the DDR. In ALT-positive cancer cells, PARP inhibitors can cause TRF2 to dissociate from telomeres, thereby stimulating the inappropriate repair of non-homologous end connections. ATM and ATR inhibitors can also be used for the treatment of ALT-positive cancers. AZD0156, an ATM inhibitor, has been used to treat ALT neuroblastomas and overcomes chemotherapy resistance (Koneru et al., 2021). As an inhibitor of the FANCM-BTR interaction (Wu et al., 2023), PIP-199 may be selectively toxic to ALT cancer cells (Lu et al., 2019), rendering it a potential therapeutic target. Tetra-Pt (bpy), a cisplatin derivative that targets telomeric G-quadruplexes, severely inhibits the growth of ALT cell xenograft tumors, indicating that it may be a novel oncotherapeutic agent for targeting ALT cancer cells (Zheng et al., 2017). In addition, bpy disrupts telomere maintenance in telomerase cancer cells, further elucidating the function of G-quadruplexes in the human genome (Shen et al., 2022). The potential of bpy as a chemotherapeutic target has been demonstrated in both ALT-positive and telomerase cancer cells.

The shelterin protein complex prevents chromosome ends from being recognised as DSB and activates the DDR (Tesmer et al., 2023). As cells divide repeatedly and telomeres continue to shorten, shelterin binding and telomere-loop (t-loop) formation are impaired, and this weakened protection leads to telomere dysfunction, cellular senescence or apoptosis (Shi et al., 2023). TRF2 overexpression has been reported to be present in a variety of malignant cancers, and its downregulation leads to cell death. Yin-da Qiu et al. showed that FKB04, a flavokawain B derivative, effectively inhibited TRF2 expression in hepatocellular carcinoma cells and also induced telomere shortening, increased the number of telomere-free ends, and led to the disruption of the T-loop structure (Qiu et al., 2024). These results suggest that TRF2 is a potential therapeutic target for hepatocellular carcinoma and indicate that FKB04 may be a selective small-molecule inhibitor of TRF2, which is expected to be used in the treatment of hepatocellular carcinoma. Mutations in TRF2 lead to changes in telomeric DNA topology, which initiates ATM-dependent DDR (Benarroch-Popivker et al., 2016). TRF1 and TRF2 form a homodimer that binds to double-stranded telomere DNA. TRF1 inhibits ATR signalling during S phase and otherwise induces a fragile telomere phenotype. TRF1 small molecule inhibitors (ETP-47228 and ETP-47037) inhibit TRF1 binding to DNA, induce DNA damage and inhibit lung cancer and glioblastoma progression, suggesting that TRF1 is a potential therapeutic target and that ETP-47228 and ETP-47037 small molecule inhibitors may be useful in treating lung cancer and glioblastoma (García-Beccaria et al., 2015; Bejarano et al., 2017). TRF2 also binds to RAP1 and inhibits the localisation of SLX4 and PARP1 to telomeres, thereby inhibiting NHEJ (Rai et al., 2016). It has been reported in the literature that Triazole-stapled peptides can block protein interactions between RAP1 and TRF2, thereby inhibiting HR (Ran et al., 2016). POT1 has been reported to co-localize with the ubiquitin-specific processing protease 7 (USP7) deubiquitinating enzyme within APB, which is capable of deubiquitinating and stabilising the POT1-targeted ubiquitin ligase, whereas testis-specific Y-coding-like protein 5 (TSPYL5) has recently been identified as a PML component and functions as a USP7 inhibitor (Episkopou et al., 2019). This suggests that TSPYL5 may be a therapeutic target for ALT-positive cancer types. The above suggests that targeting a reduction in DDR-related proteins on telomeres would reduce cancer progression. Although no reliable inhibitors are available for the above, their importance for the types of cancers in which ALT is used suggests that they should be considered in the development of future ALT-targeted therapies.

## 5 Conclusion and perspectives

Replicative immortality, a hallmark of cancer, is achieved by activating the telomere maintenance machinery (TMM), where the TMM consists of telomerase (85%–90% of tumors) and the telomere lengthening (ALT) pathway (10%–15% of tumors) (Hanahan, 2022). While telomerase inhibitors are considered promising anticancer agents, the reality is challenging; ALT cancer types are aggressive and have a poor prognosis, but no therapeutic options are currently available. Targeting telomere maintenance therefore represents an opportunity to treat the vast majority of cancer types. In this review, we identify the link between telomeres and DDR and the promising use of drugs targeting DDR therapy for the treatment of ALT cancers, and summarize recent advances in drugs targeting DDR, telomerase and ALT therapy.

**FIGURE 1:**
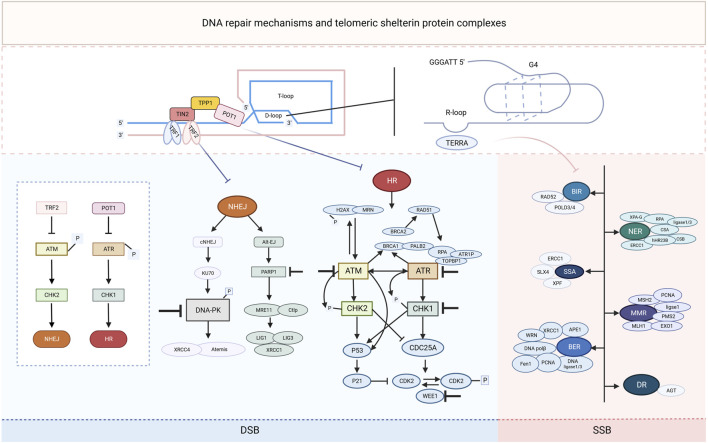
Schematic representation of DNA repair mechanisms and telomeric shelterin protein complexes. In repairing exogenous and endogenous DNA damage, cells use a range of DNA repair mechanisms, including single-strand break (SSB) and double-strand break (DSB) DNA repair pathways. SSB DNA repair mechanisms include direct repair (DR), nucleotide excision repair (NER), mismatch repair (MMR), break-induced replication (BIR), single-stranded annealing (SSA), and base excision repair (BER). The DSB response signaling is orchestrated by two kinases, ATR and ATM, which phosphorylate the substrate mainly in the G2 or G1 phases, respectively, and send signals to the cell cycle through CHK1 and CHK2. CHK2 signals cell cycle arrest and triggers both homologous recombination (HR) and non-homologous end-joining (NHEJ) DSB repair mechanisms. Each DNA repair pathway consists of a complex of signaling sources, transcription factors and effectors of the DNA repair restoration mechanism, some of the key players of which are highlighted in the figure. The telomeric shelterin protein complex inhibits the NHEJ and HR repair mechanisms, and the RNA it transcriptionally generates (TERRA) as well as the secondary structures it forms are also associated with some of the key molecules in the SSB repair mechanism. Inhibitors of these pathway components (denoted by a “T" bar) are currently in preclinical and clinical use as drugs targeting the DNA damage response.

Owing to the critical function of the DDR in cancer cells, balanced DNA damage and repair, particularly in the telomere region, is vital for cancer treatment. For example, the traditional antitumor drug, cisplatin, kills cancer cells via DNA crosstalk and induces DNA damage, which activates the ATM signaling pathway (Bian et al., 2023). Therefore, ATMi may be a potential combined drug to improve the efficacy of chemotherapy drugs. However, when considering the function of telomeres in cancer cells, the G-quadruplex ligands 20A and 6-thio-dG, which can activate ATM and/or ATR, were also confirmed to have effective antitumor activity. Therefore, the opposite effects of the same molecule should be considered in cancer treatment, which might be the key to resolving the low response rate to antitumor drugs (Figure 1).

Research on the development of drugs targeting telomeres or telomerases is ongoing. BIBR1532, MST-312, TMPyP4, RHPS4, and pyridostatin are currently undergoing preclinical research. The oligonucleotide, imetelstat, has been approved by the Food and Drug Administration for the treatment of recurrent or refractory myelofibrosis. Instead of telomerase, ALT activation in approximately 10%–15% of cancer should also be considered as a potential treatment target, particularly for DDR-related molecules.

The cGAS-STING signalling pathway is part of the innate immune system that senses both host and foreign cytosolic double-stranded DNA to initiate a type I inter feron response (Barber, 2015). Studies have shown an association between TMM and the cGAS-STING pathway, which contributes to cancer development (Ebata et al., 2022). Spontaneous immortalisation of non-malignant cells induced by TERT expression has been reported to trigger the cGAS-STING pathway, thereby altering their microenvironment to become tumour-friendly (Yang et al., 2017). ALT cancer cells have a unique feature of extrachromosomal telomere repeats (ECTR) in the cytoplasm. ECTR in normal cells activate the cGAS-STING pathway and promote immune responses leading to proliferative disorders, whereas ALT cells have a defective cGAS-STING pathway that escapes antiproliferative effects (Li et al., 2022). This suggests two major weaknesses of ALT cells. Firstly, ALT cells are able to evade ECTR-induced antiproliferative effects, but it may also lead to cells being susceptible to viral infection. Second, if the cGAS-STING pathway is active, it is potent in killing ALT cells. This suggests that testing the end product of this pathway, such as FDA-approved interferon beta (IFNβ), may be a therapeutic approach to inhibit the growth of ALT-positive cancer cells (Moglan et al., 2023). Therefore, research on the crosstalk between telomere damage and DDR is important for improving the efficacy of tumor treatment. However, further studies are required to confirm this hypothesis.

## References

[B1] Aamdal E. Inderberg E. M. Ellingsen E. B. Rasch W. Brunsvig P. F. Aamdal S. (2021). Combining a universal telomerase based cancer vaccine with ipilimumab in patients with metastatic melanoma - five-year follow up of a phase I/IIa trial. Front. Immunol. 12, 663865. 10.3389/fimmu.2021.663865 34046035 PMC8147687

[B2] Agarwal N. Azad A. A. Carles J. Fay A. P. Matsubara N. Heinrich D. (2023). Talazoparib plus enzalutamide in men with first-line metastatic castration-resistant prostate cancer (TALAPRO-2): a randomised, placebo-controlled, phase 3 trial. Lancet 402 (10398), 291–303. 10.1016/S0140-6736(23)01055-3 37285865

[B3] Alessandrini I. Percio S. Naghshineh E. Zuco V. Stacchiotti S. Gronchi A. (2022). Telomere as a therapeutic target in dedifferentiated liposarcoma. Cancers (Basel) 14 (11), 2624. 10.3390/cancers14112624 35681604 PMC9179266

[B4] Al-Karmalawy A. A. Nafie M. S. Shaldam M. A. Elmaaty A. A. Antar S. A. El-Hamaky A. A. (2023). Ligand-based design on the dog-bone-shaped BIBR1532 pharmacophoric features and synthesis of novel analogues as promising telomerase inhibitors with *in vitro* and *in vivo* evaluations. J. Med. Chem. 66 (1), 777–792. 10.1021/acs.jmedchem.2c01668 36525642

[B5] Ameri Z. Ghiasi S. Farsinejad A. Hassanshahi G. Ehsan M. Fatemi A. (2019). Telomerase inhibitor MST-312 induces apoptosis of multiple myeloma cells and down-regulation of anti-apoptotic, proliferative and inflammatory genes. Life Sci. 228, 66–71. 10.1016/j.lfs.2019.04.060 31029779

[B6] Arat N. Griffith J. D. (2012). Human Rap1 interacts directly with telomeric DNA and regulates TRF2 localization at the telomere. J. Biol. Chem. 287 (50), 41583–41594. 10.1074/jbc.M112.415984 23086976 PMC3516710

[B7] Athanasios K. Elisavet P. Eleni Kyriaki V. Despoina A. Eleftheria Kleio D. Dora H. (2014). A phase II trial evaluating the clinical and immunologic response of HLA-A2(+) non-small cell lung cancer patients vaccinated with an hTERT cryptic peptide. Lung Cancer 86 (1), 59–66. 10.1016/j.lungcan.2014.07.018 25130084

[B8] Barber G. N. (2015). STING: infection, inflammation and cancer. Nat. Rev. Immunol. 15 (12), 760–770. 10.1038/nri3921 26603901 PMC5004891

[B9] Barnes R. P. Thosar S. A. Opresko P. L. (2023). Telomere fragility and MiDAS: managing the gaps at the end of the road. Genes (Basel) 14 (2), 348. 10.3390/genes14020348 36833275 PMC9956152

[B10] Barroso-González J. García-Expósito L. Galaviz P. Lynskey M. L. Allen J. A. M. Hoang S. (2021). Anti-recombination function of MutSα restricts telomere extension by ALT-associated homology-directed repair. Cell Rep. 37 (10), 110088. 10.1016/j.celrep.2021.110088 34879271 PMC8724847

[B11] Basu B. Yap T. A. Molife L. R. de Bono J. S. (2012). Targeting the DNA damage response in oncology: past, present and future perspectives. Curr. Opin. Oncol. 24 (3), 316–324. 10.1097/CCO.0b013e32835280c6 22476188

[B12] Bayley R. Borel V. Moss R. J. Sweatman E. Ruis P. Ormrod A. (2022). H3K4 methylation by SETD1A/BOD1L facilitates RIF1-dependent NHEJ. Mol. Cell 82 (10), 1924–1939.e10. 10.1016/j.molcel.2022.03.030 35439434 PMC9616806

[B13] Beard W. A. Horton J. K. Prasad R. Wilson S. H. (2019). Eukaryotic base excision repair: new approaches shine light on mechanism. Annu. Rev. Biochem. 88, 137–162. 10.1146/annurev-biochem-013118-111315 31220977 PMC8956022

[B14] Beauvarlet J. Bensadoun P. Darbo E. Labrunie G. Rousseau B. Richard E. (2019). Modulation of the ATM/autophagy pathway by a G-quadruplex ligand tips the balance between senescence and apoptosis in cancer cells. Nucleic Acids Res. 47 (6), 2739–2756. 10.1093/nar/gkz095 30759257 PMC6451122

[B15] Bejarano L. Schuhmacher A. J. Méndez M. Megías D. Blanco-Aparicio C. Martínez S. (2017). Inhibition of TRF1 telomere protein impairs tumor initiation and progression in glioblastoma mouse models and patient-derived xenografts. Cancer Cell 32 (5), 590–607. 10.1016/j.ccell.2017.10.006 29136505

[B16] Belan O. Sebald M. Adamowicz M. Anand R. Vancevska A. Neves J. (2022). POLQ seals post-replicative ssDNA gaps to maintain genome stability in BRCA-deficient cancer cells. Mol. Cell 82 (24), 4664–4680.e9. 10.1016/j.molcel.2022.11.008 36455556

[B17] Benarroch-Popivker D. Pisano S. Mendez-Bermudez A. Lototska L. Kaur P. Bauwens S. (2016). TRF2-Mediated control of telomere DNA topology as a mechanism for chromosome-end protection. Mol. Cell 61 (2), 274–286. 10.1016/j.molcel.2015.12.009 26774283 PMC5001171

[B18] Bian H. Gu Y. Chen C. Chen J. Zhang F. Xu Z. (2023). Silence of URI in gastric cancer cells promotes cisplatin-induced DNA damage and apoptosis. Am. J. Cancer Res. 13 (3), 936–949. 37034221 PMC10077030

[B19] Biegała Ł. Gajek A. Szymczak-Pajor I. Marczak A. Śliwińska A. Rogalska A. (2023). Targeted inhibition of the ATR/CHK1 pathway overcomes resistance to olaparib and dysregulates DNA damage response protein expression in BRCA2(MUT) ovarian cancer cells. Sci. Rep. 13 (1), 22659. 10.1038/s41598-023-50151-y 38114660 PMC10730696

[B20] Blackford A. N. Jackson S. P. (2017). ATM, ATR, and DNA-PK: the trinity at the heart of the DNA damage response. Mol. Cell 66 (6), 801–817. 10.1016/j.molcel.2017.05.015 28622525

[B21] Blackwell K. Burris H. Gomez P. Lynn Henry N. Isakoff S. Campana F. (2015). Phase I/II dose-escalation study of PI3K inhibitors pilaralisib or voxtalisib in combination with letrozole in patients with hormone-receptor-positive and HER2-negative metastatic breast cancer refractory to a non-steroidal aromatase inhibitor. Breast Cancer Res. Treat. 154 (2), 287–297. 10.1007/s10549-015-3615-9 26497877

[B22] Brown J. R. Hamadani M. Hayslip J. Janssens A. Wagner-Johnston N. Ottmann O. (2018). Voxtalisib (XL765) in patients with relapsed or refractory non-Hodgkin lymphoma or chronic lymphocytic leukaemia: an open-label, phase 2 trial. Lancet Haematol. 5 (4), e170–e180. 10.1016/S2352-3026(18)30030-9 29550382 PMC7029813

[B23] Brunsvig P. F. Guren T. K. Nyakas M. Steinfeldt-Reisse C. H. Rasch W. Kyte J. A. (2020). Long-term outcomes of a phase I study with UV1, a second generation telomerase based vaccine, in patients with advanced non-small cell lung cancer. Front. Immunol. 11, 572172. 10.3389/fimmu.2020.572172 33324397 PMC7726017

[B24] Caldecott K. W. (2020). Mammalian DNA base excision repair: dancing in the moonlight. DNA Repair (Amst) 93, 102921. 10.1016/j.dnarep.2020.102921 33087262

[B25] Calvet C. Y. Thalmensi J. Liard C. Pliquet E. Bestetti T. Huet T. (2014). Optimization of a gene electrotransfer procedure for efficient intradermal immunization with an hTERT-based DNA vaccine in mice. Mol. Ther. Methods Clin. Dev. 1, 14045. 10.1038/mtm.2014.45 26015983 PMC4362362

[B26] Cavalcante S. G. Pereira B. J. A. Lerario A. M. Sola P. R. Oba-Shinjo S. M. Marie S. K. N. (2021). The chromatin remodeler complex ATRX-DAXX-H3.3 and telomere length in meningiomas. Clin. Neurol. Neurosurg. 210, 106962. 10.1016/j.clineuro.2021.106962 34624827

[B27] Cheng B. Pan W. Xing Y. Xiao Y. Chen J. Xu Z. (2022). Recent advances in DDR (DNA damage response) inhibitors for cancer therapy. Eur. J. Med. Chem. 230, 114109. 10.1016/j.ejmech.2022.114109 35051747

[B28] Chi K. N. Rathkopf D. Smith M. R. Efstathiou E. Attard G. Olmos D. (2023). Niraparib and abiraterone acetate for metastatic castration-resistant prostate cancer. J. Clin. Oncol. 41 (18), 3339–3351. 10.1200/JCO.22.01649 36952634 PMC10431499

[B29] Cleary J. M. Aguirre A. J. Shapiro G. I. D'Andrea A. D. (2020). Biomarker-guided development of DNA repair inhibitors. Mol. Cell 78 (6), 1070–1085. 10.1016/j.molcel.2020.04.035 32459988 PMC7316088

[B30] Coleman R. L. Fleming G. F. Brady M. F. Swisher E. M. Steffensen K. D. Friedlander M. (2019). Veliparib with first-line chemotherapy and as maintenance therapy in ovarian cancer. N. Engl. J. Med. 381 (25), 2403–2415. 10.1056/NEJMoa1909707 31562800 PMC6941439

[B31] Davis S. L. Hartman S. J. Bagby S. M. Schlaepfer M. Yacob B. W. Tse T. (2022). ATM kinase inhibitor AZD0156 in combination with irinotecan and 5-fluorouracil in preclinical models of colorectal cancer. BMC Cancer 22 (1), 1107. 10.1186/s12885-022-10084-7 36309653 PMC9617348

[B32] De Falco M. De F. M. (2021). Take a break to repair: a dip in the world of double-strand break repair mechanisms pointing the gaze on archaea. Int. J. Mol. Sci. 22 (24), 13296. 10.3390/ijms222413296 34948099 PMC8708640

[B33] de Lange T. (2005). Shelterin: the protein complex that shapes and safeguards human telomeres. Genes Dev. 19 (18), 2100–2110. 10.1101/gad.1346005 16166375

[B34] de Vos A. van der Helm J. Prins M. Kretzschmar M. (2012). Determinants of persistent spread of HIV in HCV-infected populations of injecting drug users. Epidemics 4 (2), 57–67. 10.1016/j.epidem.2012.01.001 22664064

[B35] Djojosubroto M. W. Chin A. C. Go N. Schaetzlein S. Manns M. P. Gryaznov S. (2005). Telomerase antagonists GRN163 and GRN163L inhibit tumor growth and increase chemosensitivity of human hepatoma. Hepatology 42 (5), 1127–1136. 10.1002/hep.20822 16114043

[B36] Doksani Y. de Lange T. (2014). The role of double-strand break repair pathways at functional and dysfunctional telomeres. Cold Spring Harb. Perspect. Biol. 6 (12), a016576. 10.1101/cshperspect.a016576 25228584 PMC4292156

[B37] Doksani Y. de Lange T. (2016). Telomere-internal double-strand breaks are repaired by homologous recombination and PARP1/lig3-dependent end-joining. Cell Rep. 17 (6), 1646–1656. 10.1016/j.celrep.2016.10.008 27806302 PMC5125555

[B38] Dos Santos G. A. Viana N. I. Pimenta R. de Camargo J. A. Guimaraes V. R. Romão P. (2024). Upregulation of shelterin and CST genes and longer telomeres are associated with unfavorable prognostic characteristics in prostate cancer. Cancer Genet. 284-285, 20–29. 10.1016/j.cancergen.2024.03.006 38503134

[B39] Eads J. R. Krishnamurthi S. S. Saltzman J. Bokar J. A. Savvides P. Meropol N. J. (2021). Phase I clinical trial of temozolomide and methoxyamine (TRC-102), an inhibitor of base excision repair, in patients with advanced solid tumors. Invest. New Drugs 39 (1), 142–151. 10.1007/s10637-020-00962-x 32556884 PMC7744386

[B40] Ebata H. Loo T. M. Takahashi A. (2022). Telomere maintenance and the cGAS-STING pathway in cancer. Cells 11 (12), 1958. 10.3390/cells11121958 35741087 PMC9221635

[B41] Elango R. Osia B. Harcy V. Malc E. Mieczkowski P. A. Roberts S. A. (2019). Repair of base damage within break-induced replication intermediates promotes kataegis associated with chromosome rearrangements. Nucleic Acids Res. 47 (18), 9666–9684. 10.1093/nar/gkz651 31392335 PMC6765108

[B42] Elango R. Sheng Z. Jackson J. DeCata J. Ibrahim Y. Pham N. T. (2017). Break-induced replication promotes formation of lethal joint molecules dissolved by Srs2. Nat. Commun. 8 (1), 1790. 10.1038/s41467-017-01987-2 29176630 PMC5702615

[B43] Episkopou H. Diman A. Claude E. Viceconte N. Decottignies A. (2019). TSPYL5 depletion induces specific death of ALT cells through USP7-dependent proteasomal degradation of POT1. Mol. Cell 75 (3), 469–482. 10.1016/j.molcel.2019.05.027 31278054

[B44] Farag N. Ercolani G. Del Grosso E. Ricci F. (2022). DNA tile self-assembly guided by base excision repair enzymes. Angew. Chem. Int. Ed. Engl. 61 (34), e202208367. 10.1002/anie.202208367 35762986

[B45] Feretzaki M. Pospisilova M. Valador Fernandes R. Lunardi T. Krejci L. Lingner J. (2020). RAD51-dependent recruitment of TERRA lncRNA to telomeres through R-loops. Nature 587 (7833), 303–308. 10.1038/s41586-020-2815-6 33057192 PMC7116795

[B46] Fernandes S. G. Gala K. Khattar E. (2022). Telomerase inhibitor MST-312 and quercetin synergistically inhibit cancer cell proliferation by promoting DNA damage. Transl. Oncol. 27, 101569. 10.1016/j.tranon.2022.101569 36274541 PMC9596868

[B47] Findlay S. Heath J. Luo V. M. Malina A. Morin T. Coulombe Y. (2018). SHLD2/FAM35A co-operates with REV7 to coordinate DNA double-strand break repair pathway choice. EMBO J. 37 (18), e100158. 10.15252/embj.2018100158 30154076 PMC6138439

[B48] Fischer-Mertens J. Otte F. Roderwieser A. Rosswog C. Kahlert Y. Werr L. (2022). Telomerase-targeting compounds Imetelstat and 6-thio-dG act synergistically with chemotherapy in high-risk neuroblastoma models. Cell Oncol. (Dordr) 45 (5), 991–1003. 10.1007/s13402-022-00702-8 35953764 PMC9579108

[B49] Fishel M. L. Xia H. McGeown J. McIlwain D. W. Elbanna M. Craft A. A. (2019). Antitumor activity and mechanistic characterization of APE1/ref-1 inhibitors in bladder cancer. Mol. Cancer Ther. 18 (11), 1947–1960. 10.1158/1535-7163.MCT-18-1166 31413178 PMC6844258

[B50] Fizazi K. Piulats J. M. Reaume M. N. Ostler P. McDermott R. Gingerich J. R. (2023). Rucaparib or physician's choice in metastatic prostate cancer. N. Engl. J. Med. 388 (8), 719–732. 10.1056/NEJMoa2214676 36795891 PMC10064172

[B51] Fu S. Yao S. Yuan Y. Previs R. A. Elias A. D. Carvajal R. D. (2023). Multicenter phase II trial of the WEE1 inhibitor adavosertib in refractory solid tumors harboring CCNE1 amplification. J. Clin. Oncol. 41 (9), 1725–1734. 10.1200/JCO.22.00830 36469840 PMC10489509

[B52] García-Beccaria M. Martínez P. Méndez-Pertuz M. Martínez S. Blanco-Aparicio C. Cañamero M. (2015). Therapeutic inhibition of TRF1 impairs the growth of p53-deficient K-RasG12V-induced lung cancer by induction of telomeric DNA damage. EMBO Mol. Med. 7 (7), 930–949. 10.15252/emmm.201404497 25971796 PMC4520658

[B53] George S. L. Parmar V. Lorenzi F. Marshall L. V. Jamin Y. Poon E. (2020). Novel therapeutic strategies targeting telomere maintenance mechanisms in high-risk neuroblastoma. J. Exp. Clin. Cancer Res. 39 (1), 78. 10.1186/s13046-020-01582-2 32375866 PMC7201617

[B54] Gerada C. Campbell T. M. Kennedy J. J. McSharry B. P. Steain M. Slobedman B. (2020). Manipulation of the innate immune response by varicella zoster virus. Front. Immunol. 11, 1. 10.3389/fimmu.2020.00001 32038653 PMC6992605

[B55] Gomez D. E. Armando R. G. Alonso D. F. (2012). AZT as a telomerase inhibitor. Front. Oncol. 2, 113. 10.3389/fonc.2012.00113 22973556 PMC3434370

[B56] Gómez-Cabello D. Pappas G. Aguilar-Morante D. Dinant C. Bartek J. (2022). CtIP-dependent nascent RNA expression flanking DNA breaks guides the choice of DNA repair pathway. Nat. Commun. 13 (1), 5303. 10.1038/s41467-022-33027-z 36085345 PMC9463442

[B57] Gu P. Chang S. (2013). Functional characterization of human CTC1 mutations reveals novel mechanisms responsible for the pathogenesis of the telomere disease Coats plus. Aging Cell 12 (6), 1100–1109. 10.1111/acel.12139 23869908 PMC4083614

[B58] Guh C. Y. Shen H. J. Chen L. W. Chiu P. C. Liao I. H. Lo C. C. (2022). XPF activates break-induced telomere synthesis. Nat. Commun. 13 (1), 5781. 10.1038/s41467-022-33428-0 36184605 PMC9527253

[B59] Haakensen V. D. Öjlert Å. K. Thunold S. Farooqi S. Nowak A. K. Chin W. L. (2024). UV1 telomerase vaccine with ipilimumab and nivolumab as second line treatment for pleural mesothelioma - a phase II randomised trial. Eur. J. Cancer 202, 113973. 10.1016/j.ejca.2024.113973 38447379

[B60] Hanahan D. (2022). Hallmarks of cancer: new dimensions. Cancer Discov. 12 (1), 31–46. 10.1158/2159-8290.CD-21-1059 35022204

[B61] Harold J. Bellone S. Manavella D. D. Mutlu L. McNamara B. Hartwich T. M. P. (2023). Elimusertib (BAY1895344), a novel ATR inhibitor, demonstrates *in vivo* activity in ATRX mutated models of uterine leiomyosarcoma. Gynecol. Oncol. 168, 157–165. 10.1016/j.ygyno.2022.11.014 36442427 PMC9797429

[B62] Hernandez-Sanchez W. Huang W. Plucinsky B. Garcia-Vazquez N. Robinson N. J. Schiemann W. P. (2019). A non-natural nucleotide uses a specific pocket to selectively inhibit telomerase activity. PLoS Biol. 17 (4), e3000204. 10.1371/journal.pbio.3000204 30951520 PMC6469803

[B63] Hu X. Hsieh C. Y. Zhang Y. Liu W. Xu S. Cai S. X. (2023). Effect of a strong CYP3A4 inhibitor and inducer on the pharmacokinetics of senaparib (IMP4297) in healthy volunteers: a drug-drug interaction study. Br. J. Clin. Pharmacol. 89 (6), 1767–1779. 10.1111/bcp.15624 36458825

[B64] Huang P. Q. Boren B. C. Hegde S. G. Liu H. Unni A. K. Abraham S. (2021). Discovery of ZN-c3, a highly potent and selective Wee1 inhibitor undergoing evaluation in clinical trials for the treatment of cancer. J. Med. Chem. 64 (17), 13004–13024. 10.1021/acs.jmedchem.1c01121 34423975

[B65] Huffman B. M. Feng H. Parmar K. Wang J. Kapner K. S. Kochupurakkal B. (2023). A phase I expansion cohort study evaluating the safety and efficacy of the CHK1 inhibitor LY2880070 with low-dose gemcitabine in patients with metastatic pancreatic adenocarcinoma. Clin. Cancer Res. 29 (24), 5047–5056. 10.1158/1078-0432.CCR-23-2005 37819936 PMC10842136

[B66] Iida K. Suzuki N. Sasaki A. Ishida S. Arai T. (2022). Development of a novel light-up probe for detection of G-quadruplexes in stress granules. Sci. Rep. 12 (1), 12892. 10.1038/s41598-022-17230-y 35902691 PMC9334577

[B67] Illuzzi G. Staniszewska A. Gill S. Pike A. McWilliams L. Critchlow S. (2022). Preclinical characterization of AZD5305, A next-generation, highly selective PARP1 inhibitor and trapper. Clin. cancer Res. 28 (21), 4724–4736. 10.1158/1078-0432.CCR-22-0301 35929986 PMC9623235

[B68] Infante J. R. Hollebecque A. Postel-Vinay S. Bauer T. M. Blackwood E. M. Evangelista M. (2017). Phase I study of GDC-0425, a checkpoint kinase 1 inhibitor, in combination with gemcitabine in patients with refractory solid tumors. Clin. Cancer Res. 23 (10), 2423–2432. 10.1158/1078-0432.CCR-16-1782 27815358

[B69] Kaminski N. Wondisford A. R. Kwon Y. Lynskey M. L. Bhargava R. Barroso-González J. (2022). RAD51AP1 regulates ALT-HDR through chromatin-directed homeostasis of TERRA. Mol. Cell 82 (21), 4001–4017.e7. 10.1016/j.molcel.2022.09.025 36265488 PMC9713952

[B70] Kaul Z. Cheung C. T. Y. Bhargava P. Sari A. N. Yu Y. Huifu H. (2021). Functional characterization of miR-708 microRNA in telomerase positive and negative human cancer cells. Sci. Rep. 11 (1), 17052. 10.1038/s41598-021-96096-y 34426596 PMC8382839

[B71] Kim D. E. Dollé M. E. T. Vermeij W. P. Gyenis A. Vogel K. Hoeijmakers J. H. J. (2020). Deficiency in the DNA repair protein ERCC1 triggers a link between senescence and apoptosis in human fibroblasts and mouse skin. Aging Cell 19 (3), e13072. 10.1111/acel.13072 31737985 PMC7059167

[B72] Kim H. Seo E. H. Lee S. H. Kim B. J. (2016). The telomerase-derived anticancer peptide vaccine GV1001 as an extracellular heat shock protein-mediated cell-penetrating peptide. Int. J. Mol. Sci. 17 (12), 2054. 10.3390/ijms17122054 27941629 PMC5187854

[B73] Kim R. Kwon M. An M. Kim S. Smith S. Loembé A. (2022). Phase II study of ceralasertib (AZD6738) in combination with durvalumab in patients with advanced/metastatic melanoma who have failed prior anti-PD-1 therapy. Ann. Oncol. 33 (2), 193–203. 10.1016/j.annonc.2021.10.009 34710570

[B74] Kishkevich A. Tamang S. Nguyen M. O. Oehler J. Bulmaga E. Andreadis C. (2022). Rad52's DNA annealing activity drives template switching associated with restarted DNA replication. Nat. Commun. 13 (1), 7293. 10.1038/s41467-022-35060-4 36435847 PMC9701231

[B75] Kockler Z. W. Osia B. Lee R. Musmaker K. Malkova A. (2021). Repair of DNA breaks by break-induced replication. Annu. Rev. Biochem. 90, 165–191. 10.1146/annurev-biochem-081420-095551 33792375 PMC9629446

[B76] Kodym E. Kodym R. Reis A. Habib A. Story M. Saha D. (2009). The small-molecule CDK inhibitor, SNS-032, enhances cellular radiosensitivity in quiescent and hypoxic non-small cell lung cancer cells. Lung cancer (Amsterdam, Neth.) 66 (1), 37–47. 10.1016/j.lungcan.2008.12.026 19193471

[B77] Koneru B. Farooqi A. Nguyen T. H. Chen W. H. Hindle A. Eslinger C. (2021). ALT neuroblastoma chemoresistance due to telomere dysfunction-induced ATM activation is reversible with ATM inhibitor AZD0156. Sci. Transl. Med. 13 (607), eabd5750. 10.1126/scitranslmed.abd5750 34408079 PMC9208664

[B78] Konstantinopoulos P. A. Lee J. M. Gao B. Miller R. Lee J. Y. Colombo N. (2022). A Phase 2 study of prexasertib (LY2606368) in platinum resistant or refractory recurrent ovarian cancer. Gynecol. Oncol. 167 (2), 213–225. 10.1016/j.ygyno.2022.09.019 36192237 PMC10673677

[B79] Kreuzer K. N. (2000). Recombination-dependent DNA replication in phage T4. Trends Biochem. Sci. 25 (4), 165–173. 10.1016/s0968-0004(00)01559-0 10754548

[B80] Kunkel T. A. Erie D. A. (2015). Eukaryotic mismatch repair in relation to DNA replication. Annu. Rev. Genet. 49, 291–313. 10.1146/annurev-genet-112414-054722 26436461 PMC5439269

[B81] Li X. Li X. Xie C. Cai S. Li M. Jin H. (2022). cGAS guards against chromosome end-to-end fusions during mitosis and facilitates replicative senescence. Protein Cell 13 (1), 47–64. 10.1007/s13238-021-00879-y 34676498 PMC8776970

[B82] Lilleby W. Gaudernack G. Brunsvig P. F. Vlatkovic L. Schulz M. Mills K. (2017). Phase I/IIa clinical trial of a novel hTERT peptide vaccine in men with metastatic hormone-naive prostate cancer. Cancer Immunol. Immunother. 66 (7), 891–901. 10.1007/s00262-017-1994-y 28391357 PMC11028648

[B83] Liu B. He Y. Wang Y. Song H. Zhou Z. Feigon J. (2022a). Structure of active human telomerase with telomere shelterin protein TPP1. Nature 604 (7906), 578–583. 10.1038/s41586-022-04582-8 35418675 PMC9912816

[B84] Liu L. Y. Ma T. Z. Zeng Y. L. Liu W. Mao Z. W. (2022b). Structural basis of pyridostatin and its derivatives specifically binding to G-quadruplexes. J. Am. Chem. Soc. 144 (26), 11878–11887. 10.1021/jacs.2c04775 35749293

[B85] Lu R. O'Rourke J. J. Sobinoff A. P. Allen J. A. M. Nelson C. B. Tomlinson C. G. (2019). The FANCM-BLM-TOP3A-RMI complex suppresses alternative lengthening of telomeres (ALT). Nat. Commun. 10 (1), 2252. 10.1038/s41467-019-10180-6 31138797 PMC6538672

[B86] Luedeman M. E. Stroik S. Feng W. Luthman A. J. Gupta G. P. Ramsden D. A. (2022). Poly(ADP) ribose polymerase promotes DNA polymerase theta-mediated end joining by activation of end resection. Nat. Commun. 13 (1), 4547. 10.1038/s41467-022-32166-7 35927262 PMC9352658

[B87] Luo Z. Liu W. Sun P. Wang F. Feng X. (2021). Pan-cancer analyses reveal regulation and clinical outcome association of the shelterin complex in cancer. Brief. Bioinform 22 (5), bbaa441. 10.1093/bib/bbaa441 33497432

[B88] Maréchal A. Zou L. (2013). DNA damage sensing by the ATM and ATR kinases. Cold Spring Harb. Perspect. Biol. 5 (9), a012716. 10.1101/cshperspect.a012716 24003211 PMC3753707

[B89] Mender I. LaRanger R. Luitel K. Peyton M. Girard L. Lai T. (2018). Telomerase-mediated strategy for overcoming non-small cell lung cancer targeted therapy and chemotherapy resistance. Neoplasia (New York, NY) 20 (8), 826–837. 10.1016/j.neo.2018.06.002 PMC6037876 30015158

[B90] Mender I. Zhang A. Ren Z. Han C. Deng Y. Siteni S. (2020). Telomere stress potentiates STING-dependent anti-tumor immunity. Cancer Cell 38 (3), 400–411. 10.1016/j.ccell.2020.05.020 32619407 PMC7494563

[B91] Menez-Jamet J. Gallou C. Rougeot A. Kosmatopoulos K. (2016). Optimized tumor cryptic peptides: the basis for universal neo-antigen-like tumor vaccines. Ann. Transl. Med. 4 (14), 266. 10.21037/atm.2016.05.15 27563653 PMC4971378

[B92] Middleton G. Silcocks P. Cox T. Valle J. Wadsley J. Propper D. (2014). Gemcitabine and capecitabine with or without telomerase peptide vaccine GV1001 in patients with locally advanced or metastatic pancreatic cancer (TeloVac): an open-label, randomised, phase 3 trial. Lancet Oncol. 15 (8), 829–840. 10.1016/S1470-2045(14)70236-0 24954781

[B93] Moglan A. M. Albaradie O. A. Alsayegh F. F. Alharbi H. M. Samman Y. M. Jalal M. M. (2023). Preclinical efficacy of oncolytic VSV-IFNβ in treating cancer: a systematic review. Front. Immunol. 14, 1085940. 10.3389/fimmu.2023.1085940 37063914 PMC10104167

[B94] Mukherjee J. Johannessen T. C. Ohba S. Chow T. T. Jones L. Pandita A. (2018). Mutant IDH1 cooperates with ATRX loss to drive the alternative lengthening of telomere phenotype in glioma. Cancer Res. 78 (11), 2966–2977. 10.1158/0008-5472.CAN-17-2269 29545335 PMC10578296

[B95] Murciano-Goroff Y. Schram A. Rosen E. Won H. Gong Y. Noronha A. (2022). Reversion mutations in germline BRCA1/2-mutant tumors reveal a BRCA-mediated phenotype in non-canonical histologies. Nat. Commun. 13 (1), 7182. 10.1038/s41467-022-34109-8 36418296 PMC9684575

[B96] Nagel Z. D. Kitange G. J. Gupta S. K. Joughin B. A. Chaim I. A. Mazzucato P. (2017). DNA repair capacity in multiple pathways predicts chemoresistance in glioblastoma multiforme. Cancer Res. 77 (1), 198–206. 10.1158/0008-5472.CAN-16-1151 27793847 PMC6100738

[B97] Ngo G. H. P. Grimstead J. W. Baird D. M. (2021). UPF1 promotes the formation of R loops to stimulate DNA double-strand break repair. Nat. Commun. 12 (1), 3849. 10.1038/s41467-021-24201-w 34158508 PMC8219777

[B98] Oanh N. T. K. Lee H. S. Kim Y. H. Min S. Park Y. J. Heo J. (2022). Regulation of nuclear DNA damage response by mitochondrial morphofunctional pathway. Nucleic Acids Res. 50 (16), 9247–9259. 10.1093/nar/gkac690 35979947 PMC9458461

[B99] Ouyang J. Garner E. Hallet A. Nguyen H. D. Rickman K. A. Gill G. (2015). Noncovalent interactions with SUMO and ubiquitin orchestrate distinct functions of the SLX4 complex in genome maintenance. Mol. Cell 57 (1), 108–122. 10.1016/j.molcel.2014.11.015 25533185 PMC4289429

[B100] Park S. J. Chang S. J. Suh D. H. Kong T. W. Song H. Kim T. H. (2022). A phase IA dose-escalation study of PHI-101, a new checkpoint kinase 2 inhibitor, for platinum-resistant recurrent ovarian cancer. BMC Cancer 22 (1), 28. 10.1186/s12885-021-09138-z 34980026 PMC8722005

[B101] Peng X. Zhang S. Wang Y. Zhou Z. Yu Z. Zhong Z. (2023). Stellettin B sensitizes glioblastoma to DNA-damaging treatments by suppressing PI3K-mediated homologous recombination repair. Adv. Sci. (Weinh) 10 (3), e2205529. 10.1002/advs.202205529 36453577 PMC9875605

[B102] Perkhofer L. Gout J. Roger E. Kude de Almeida F. Baptista Simões C. Wiesmüller L. (2021). DNA damage repair as a target in pancreatic cancer: state-of-the-art and future perspectives. Gut 70 (3), 606–617. 10.1136/gutjnl-2019-319984 32855305 PMC7873425

[B103] Pfitzer L. Moser C. Gegenfurtner F. Arner A. Foerster F. Atzberger C. (2019). Targeting actin inhibits repair of doxorubicin-induced DNA damage: a novel therapeutic approach for combination therapy. Cell Death Dis. 10 (4), 302. 10.1038/s41419-019-1546-9 30944311 PMC6447524

[B104] Pobiega S. Alibert O. Marcand S. (2021). A new assay capturing chromosome fusions shows a protection trade-off at telomeres and NHEJ vulnerability to low-density ionizing radiation. Nucleic Acids Res. 49 (12), 6817–6831. 10.1093/nar/gkab502 34125900 PMC8266670

[B105] Qin H. Guo Y. (2022). Targeting telomerase enhances cytotoxicity of salinomycin in cancer cells. ACS Omega 7 (34), 30565–30570. 10.1021/acsomega.2c04082 36061682 PMC9435028

[B106] Qin T. Mullan B. Ravindran R. Messinger D. Siada R. Cummings J. R. (2022). ATRX loss in glioma results in dysregulation of cell-cycle phase transition and ATM inhibitor radio-sensitization. Cell Rep. 38 (2), 110216. 10.1016/j.celrep.2021.110216 35021084 PMC8759735

[B107] Qiu Y. D. Yan Q. Wang Y. Ye Y. F. Wang Y. Wang M. Y. (2024). Discovery of a selective TRF2 inhibitor FKB04 induced telomere shortening and senescence in liver cancer cells. Acta Pharmacol. Sin. 10.1038/s41401-024-01243-6 PMC11130216 38438580

[B108] Radin D. P. Shifman S. Outhwaite I. R. Sharma A. Bases R. Seeliger M. A. (2024). Lucanthone, a potential PPT1 inhibitor, perturbs stemness, reduces tumor microtube formation, and slows the growth of temozolomide-resistant gliomas *in vivo* . J. Pharmacol. Exp. Ther. 389 (1), 51–60. 10.1124/jpet.123.002021 38296645 PMC10949164

[B109] Raghunandan M. Geelen D. Majerova E. Decottignies A. (2021). NHP2 downregulation counteracts hTR-mediated activation of the DNA damage response at ALT telomeres. Embo J. 40 (6), e106336. 10.15252/embj.2020106336 33595114 PMC7957427

[B110] Rai R. Chen Y. Lei M. Chang S. (2016). TRF2-RAP1 is required to protect telomeres from engaging in homologous recombination-mediated deletions and fusions. Nat. Commun. 7, 10881. 10.1038/ncomms10881 26941064 PMC4785230

[B111] Ran X. Liu L. Yang C. Y. Lu J. Chen Y. Lei M. (2016). Design of high-affinity stapled peptides to target the repressor activator protein 1 (RAP1)/Telomeric repeat-binding factor 2 (TRF2) protein-protein interaction in the shelterin complex. J. Med. Chem. 59 (1), 328–334. 10.1021/acs.jmedchem.5b01465 26673461

[B112] Ribes-Zamora A. Indiviglio S. M. Mihalek I. Williams C. L. Bertuch A. A. (2013). TRF2 interaction with Ku heterotetramerization interface gives insight into c-NHEJ prevention at human telomeres. Cell Rep. 5 (1), 194–206. 10.1016/j.celrep.2013.08.040 24095731 PMC3984498

[B113] Robson M. Im S. A. Senkus E. Xu B. Domchek S. M. Masuda N. (2017). Olaparib for metastatic breast cancer in patients with a germline BRCA mutation. N. Engl. J. Med. 377 (6), 523–533. 10.1056/NEJMoa1706450 28578601

[B114] Roos W. P. Thomas A. D. Kaina B. (2016). DNA damage and the balance between survival and death in cancer biology. Nat. Rev. Cancer 16 (1), 20–33. 10.1038/nrc.2015.2 26678314

[B115] Saito T. T. Youds J. L. Boulton S. J. Colaiácovo M. P. (2009). *Caenorhabditis elegans* HIM-18/SLX-4 interacts with SLX-1 and XPF-1 and maintains genomic integrity in the germline by processing recombination intermediates. PLoS Genet. 5 (11), e1000735. 10.1371/journal.pgen.1000735 19936019 PMC2770170

[B116] Sakellariou D. Bak S. T. Isik E. Barroso S. I. Porro A. Aguilera A. (2022). MutSβ regulates G4-associated telomeric R-loops to maintain telomere integrity in ALT cancer cells. Cell Rep. 39 (1), 110602. 10.1016/j.celrep.2022.110602 35385755

[B117] Samuels M. Falkenius J. Bar-Ad V. Dunst J. van Triest B. Yachnin J. (2024). A phase 1 study of the DNA-PK inhibitor peposertib in combination with radiation therapy with or without cisplatin in patients with advanced head and neck tumors. Int. J. Radiat. Oncol. Biol. Phys. 118 (3), 743–756. 10.1016/j.ijrobp.2023.09.024 37751793

[B118] Scheper J. Hildebrand L. S. Faulhaber E. M. Deloch L. Gaipl U. S. Symank J. (2022). Tumor-specific radiosensitizing effect of the ATM inhibitor AZD0156 in melanoma cells with low toxicity to healthy fibroblasts. Strahlenther Onkol. 199, 1128–1139. 10.1007/s00066-022-02009-x 36229655 PMC10673781

[B119] Schutte T. Embaby A. Steeghs N. van der Mierden S. van Driel W. Rijlaarsdam M. (2023). Clinical development of WEE1 inhibitors in gynecological cancers: a systematic review. Cancer Treat. Rev. 115, 102531. 10.1016/j.ctrv.2023.102531 36893690

[B120] Scully R. Panday A. Elango R. Willis N. A. (2019). DNA double-strand break repair-pathway choice in somatic mammalian cells. Nat. Rev. Mol. Cell Biol. 20 (11), 698–714. 10.1038/s41580-019-0152-0 31263220 PMC7315405

[B121] Selvaraj S. Feist W. N. Viel S. Vaidyanathan S. Dudek A. M. Gastou M. (2023). High-efficiency transgene integration by homology-directed repair in human primary cells using DNA-PKcs inhibition. Nat. Biotechnol. 42, 731–744. 10.1038/s41587-023-01888-4 37537500

[B122] Sengupta S. Sobo M. Lee K. Senthil Kumar S. White A. R. Mender I. (2018). Induced telomere damage to treat telomerase expressing therapy-resistant pediatric brain tumors. Mol. Cancer Ther. 17 (7), 1504–1514. 10.1158/1535-7163.MCT-17-0792 29654065

[B123] Shankar U. Mishra S. K. Jain N. Tawani A. Yadav P. Kumar A. (2022). Ni(+2) permease system of *Helicobacter pylori* contains highly conserved G-quadruplex motifs. Infect. Genet. Evol. 101, 105298. 10.1016/j.meegid.2022.105298 35526824

[B124] Shen Z. Zheng R. Yang H. Xing S. Jin X. Yan H. (2022). G-quadruplex stabilizer Tetra-Pt(bpy) disrupts telomere maintenance and impairs FAK-mediated migration of telomerase-positive cells. Int. J. Biol. Macromol. 213, 858–870. 10.1016/j.ijbiomac.2022.06.015 35697164

[B125] Shi J. Zhang M. Zhang L. Yu X. Sun L. Liu J. (2023). Shelterin dysfunction promotes CD4+ T cell senescence in behçet's disease. Rheumatol. Oxf., kead703. 10.1093/rheumatology/kead703 38145496

[B126] Shibata A. Jeggo P. A. (2020). Canonical DNA non-homologous end-joining; capacity versus fidelity. Br. J. Radiol. 93 (1115), 20190966. 10.1259/bjr.20190966 31944860 PMC8519634

[B127] Silva B. Arora R. Bione S. Azzalin C. M. (2021). TERRA transcription destabilizes telomere integrity to initiate break-induced replication in human ALT cells. Nat. Commun. 12 (1), 3760. 10.1038/s41467-021-24097-6 34145295 PMC8213692

[B128] Silva B. Pentz R. Figueira A. M. Arora R. Lee Y. W. Hodson C. (2019). FANCM limits ALT activity by restricting telomeric replication stress induced by deregulated BLM and R-loops. Nat. Commun. 10 (1), 2253. 10.1038/s41467-019-10179-z 31138795 PMC6538666

[B129] Slijepcevic P. (2006). The role of DNA damage response proteins at telomeres-an "integrative" model. DNA Repair (Amst). 5 (11), 1299–1306. 10.1016/j.dnarep.2006.05.038 16798109

[B130] Somuncu B. Keskin S. Antmen F. M. Saglican Y. Ekmekcioglu A. Ertuzun T. (2020). Non-muscle invasive bladder cancer tissues have increased base excision repair capacity. Sci. Rep. 10 (1), 16371. 10.1038/s41598-020-73370-z 33004944 PMC7529820

[B131] Stundon J. L. Ijaz H. Gaonkar K. S. Kaufman R. S. Jin R. Karras A. (2022). ALT in pediatric high-grade gliomas can occur without ATRX mutation and is enriched in patients with pathogenic germline MMR variants OGG1 in the kidney: beyond base excision repair. Neuro Oncol. 2022, 5774641. 10.1093/neuonc/noac278 PMC10326481 36541551

[B132] Subecz C. Sun J. S. Roger L. (2021). Effect of DNA repair inhibitor AsiDNA on the incidence of telomere fusion in crisis. Hum. Mol. Genet. 30 (3-4), 172–181. 10.1093/hmg/ddab008 33480989 PMC8091035

[B133] Sweeney C. J. Percent I. J. Babu S. Cultrera J. L. Mehlhaff B. A. Goodman O. B. (2022). Phase ib/II study of enzalutamide with samotolisib (LY3023414) or placebo in patients with metastatic castration-resistant prostate cancer. Clin. Cancer Res. 28 (11), 2237–2247. 10.1158/1078-0432.CCR-21-2326 35363301 PMC9662871

[B134] Szymanski M. R. Karlowicz A. Herrmann G. K. Cen Y. Yin Y. W. (2022). Human EXOG possesses strong AP hydrolysis activity: implication on mitochondrial DNA base excision repair. J. Am. Chem. Soc. 144 (51), 23543–23550. 10.1021/jacs.2c10558 36516439 PMC10920074

[B135] Takahashi N. Hao Z. Villaruz L. C. Zhang J. Ruiz J. Petty W. J. (2023). Berzosertib plus topotecan vs topotecan alone in patients with relapsed small cell lung cancer: a randomized clinical trial. JAMA Oncol. 9 (12), 1669–1677. 10.1001/jamaoncol.2023.4025 37824137 PMC10570917

[B136] Tang Q. Gulkis M. McKenna R. Çağlayan M. (2022). Structures of LIG1 that engage with mutagenic mismatches inserted by polβ in base excision repair. Nat. Commun. 13 (1), 3860. 10.1038/s41467-022-31585-w 35790757 PMC9256674

[B137] Teixeira L. Medioni J. Garibal J. Adotevi O. Doucet L. Durey M. D. (2020). A first-in-human phase I study of INVAC-1, an optimized human telomerase DNA vaccine in patients with advanced solid tumors. Clin. Cancer Res. 26 (3), 588–597. 10.1158/1078-0432.CCR-19-1614 31558479

[B138] Telli M. L. Tolaney S. M. Shapiro G. I. Middleton M. Lord S. R. Arkenau H. T. (2022). Phase 1b study of berzosertib and cisplatin in patients with advanced triple-negative breast cancer. NPJ Breast Cancer 8 (1), 45. 10.1038/s41523-022-00406-0 35393425 PMC8991212

[B139] Teng F. Y. Jiang Z. Z. Guo M. Tan X. Z. Chen F. Xi X. G. (2021). G-quadruplex DNA: a novel target for drug design. Cell Mol. Life Sci. 78 (19-20), 6557–6583. 10.1007/s00018-021-03921-8 34459951 PMC11072987

[B140] Tesmer V. M. Brenner K. A. Nandakumar J. (2023). Human POT1 protects the telomeric ds-ss DNA junction by capping the 5' end of the chromosome. Science 381 (6659), 771–778. 10.1126/science.adi2436 37590346 PMC10666826

[B141] Tiek D. Erdogdu B. Razaghi R. Jin L. Sadowski N. Alamillo-Ferrer C. (2022). Temozolomide-induced guanine mutations create exploitable vulnerabilities of guanine-rich DNA and RNA regions in drug-resistant gliomas. Sci. Adv. 8 (25), eabn3471. 10.1126/sciadv.abn3471 35731869 PMC9216507

[B142] Tsai R. Fang K. Yang P. Hsieh Y. Chiang I. Chen Y. (2022). TERRA regulates DNA G-quadruplex formation and ATRX recruitment to chromatin. Nucleic acids Res. 50 (21), 12217–12234. 10.1093/nar/gkac1114 36440760 PMC9757062

[B143] Ueno M. (2023). Exploring genetic interactions with telomere protection gene pot1 in fission yeast. Biomolecules 13 (2), 370. 10.3390/biom13020370 36830739 PMC9953254

[B144] Vancevska A. Ahmed W. Pfeiffer V. Feretzaki M. Boulton S. J. Lingner J. (2020). SMCHD1 promotes ATM-dependent DNA damage signaling and repair of uncapped telomeres. EMBO J. 39 (7), e102668. 10.15252/embj.2019102668 32080884 PMC7110143

[B145] van de Kooij B. Kruswick A. van Attikum H. Yaffe M. B. (2022). Multi-pathway DNA-repair reporters reveal competition between end-joining, single-strand annealing and homologous recombination at Cas9-induced DNA double-strand breaks. Nat. Commun. 13 (1), 5295. 10.1038/s41467-022-32743-w 36075911 PMC9458747

[B146] van Harten A. M. Buijze M. van der Mast R. Rooimans M. A. Martens-de Kemp S. R. Bachas C. (2019). Targeting the cell cycle in head and neck cancer by Chk1 inhibition: a novel concept of bimodal cell death. Oncogenesis 8 (7), 38. 10.1038/s41389-019-0147-x 31209198 PMC6572811

[B147] Vassilis G. Jean-Yves D. David K. Christian M. Rafael R. Antonio R. (2013). A multicenter randomized phase IIb efficacy study of Vx-001, a peptide-based cancer vaccine as maintenance treatment in advanced non-small-cell lung cancer: treatment rationale and protocol dynamics. Clin. Lung Cancer 14 (4), 461–465. 10.1016/j.cllc.2013.02.001 23647738

[B148] Vendetti F. P. Karukonda P. Clump D. A. Teo T. Lalonde R. Nugent K. (2018). ATR kinase inhibitor AZD6738 potentiates CD8+ T cell-dependent antitumor activity following radiation. J. Clin. Invest. 128 (9), 3926–3940. 10.1172/JCI96519 29952768 PMC6118586

[B149] Verdun R. E. Karlseder J. (2007). Replication and protection of telomeres. Nature 447 (7147), 924–931. 10.1038/nature05976 17581575

[B150] Verma P. Dilley R. L. Zhang T. Gyparaki M. T. Li Y. Greenberg R. A. (2019). RAD52 and SLX4 act nonepistatically to ensure telomere stability during alternative telomere lengthening. Genes Dev. 33 (3-4), 221–235. 10.1101/gad.319723.118 30692206 PMC6362809

[B151] Vernì F. (2022). DNA damage response (DDR) and DNA repair. Int. J. Mol. Sci. 23 (13), 7204. 10.3390/ijms23137204 35806207 PMC9266642

[B152] Viol F. Sipos B. Fahl M. Clauditz T. S. Amin T. Kriegs M. (2022). Novel preclinical gastroenteropancreatic neuroendocrine neoplasia models demonstrate the feasibility of mutation-based targeted therapy. Cell Oncol. (Dordr) 45 (6), 1401–1419. 10.1007/s13402-022-00727-z 36269546 PMC9747820

[B153] Vohhodina J. Goehring L. J. Liu B. Kong Q. Botchkarev V. V. Huynh M. (2021). BRCA1 binds TERRA RNA and suppresses R-Loop-based telomeric DNA damage. Nat. Commun. 12 (1), 3542. 10.1038/s41467-021-23716-6 34112789 PMC8192922

[B154] Wen P. Y. Omuro A. Ahluwalia M. S. Fathallah-Shaykh H. M. Mohile N. Lager J. J. (2015). Phase I dose-escalation study of the PI3K/mTOR inhibitor voxtalisib (SAR245409, XL765) plus temozolomide with or without radiotherapy in patients with high-grade glioma. Neuro Oncol. 17 (9), 1275–1283. 10.1093/neuonc/nov083 26019185 PMC4588757

[B155] Wong W. K. Guerra Liberal F. D. C. McMahon S. J. (2022). DNA repair inhibitors potentiate fractionated radiotherapy more than single-dose radiotherapy in breast cancer cells. Cancers (Basel) 14 (15), 3794. 10.3390/cancers14153794 35954456 PMC9367425

[B156] Wu J. Dai W. Wu L. Wang J. (2018). SALP, a new single-stranded DNA library preparation method especially useful for the high-throughput characterization of chromatin openness states. BMC Genomics 19 (1), 143. 10.1186/s12864-018-4530-3 29439663 PMC5811972

[B157] Wu L. Fidan K. Um J. Y. Ahn K. S. (2020). Telomerase: key regulator of inflammation and cancer. Pharmacol. Res. 155, 104726. 10.1016/j.phrs.2020.104726 32109579

[B158] Wu X. Krishna Sudhakar H. Alcock L. J. Lau Y. H. (2023). Mannich base PIP-199 is a chemically unstable pan-assay interference compound. J. Med. Chem. 66 (16), 11271–11281. 10.1021/acs.jmedchem.3c00674 37555818

[B159] Xue H. Bhardwaj A. Yin Y. Fijen C. Ephstein A. Zhang L. (2022). A two-step mechanism governing PARP1-DNA retention by PARP inhibitors. Sci. Adv. 8 (36), eabq0414. 10.1126/sciadv.abq0414 36070389 PMC9451145

[B160] Yadav T. Zhang J. M. Ouyang J. Leung W. Simoneau A. Zou L. (2022). TERRA and RAD51AP1 promote alternative lengthening of telomeres through an R-to D-loop switch. Mol. Cell 82 (21), 3985–4000.e4. 10.1016/j.molcel.2022.09.026 36265486 PMC9637728

[B161] Yan C. Chang Y. Gao H. Zhang Q. Peng S. Wang D. (2021). G-quadruplex apurinic site-programmed chiral cyanine assemblies for specifically recognizing guanosine and guanine. Analyst 146 (19), 5866–5872. 10.1039/d1an01110c 34570847

[B162] Yang H. Wang H. Ren J. Chen Q. Chen Z. J. (2017). cGAS is essential for cellular senescence. Proc. Natl. Acad. Sci. U. S. A. 114 (23), E4612–e20. 10.1073/pnas.1705499114 28533362 PMC5468617

[B163] Yang Z. Sharma K. de Lange T. (2022). TRF1 uses a noncanonical function of TFIIH to promote telomere replication. Genes Dev. 36 (17-18), 956–969. 10.1101/gad.349975.122 36229075 PMC9732906

[B164] Yap T. A. Fontana E. Lee E. K. Spigel D. R. Højgaard M. Lheureux S. (2023). Camonsertib in DNA damage response-deficient advanced solid tumors: phase 1 trial results. Nat. Med. 29 (6), 1400–1411. 10.1038/s41591-023-02399-0 37277454 PMC10287555

[B165] Yap T. A. Tolcher A. W. Plummer R. Mukker J. K. Enderlin M. Hicking C. (2024). First-in-Human study of the ataxia telangiectasia and rad3-related (ATR) inhibitor tuvusertib (M1774) as monotherapy in patients with solid tumors. Clin. Cancer Res. 30, 2057–2067. 10.1158/1078-0432.CCR-23-2409 38407317 PMC11094421

[B166] Yilmaz U. Kamer D. Asik A. Kara H. G. Gündüz C. Kamer S. (2023). The effect of the ATM inhibitor AZD0156 on the radiosensitivity of human breast cancer and lung fibroblast cells. J. Cancer Res. Ther. 19 (2), 203–207. 10.4103/jcrt.JCRT_1540_20 37006058

[B167] Yu S. Wei S. Savani M. Lin X. Du K. Mender I. (2021). A modified nucleoside 6-thio-2'-deoxyguanosine exhibits antitumor activity in gliomas. Clin. Cancer Res. 27 (24), 6800–6814. 10.1158/1078-0432.CCR-21-0374 34593527 PMC8678347

[B168] Zhang J. M. Genois M. M. Ouyang J. Lan L. Zou L. (2021). Alternative lengthening of telomeres is a self-perpetuating process in ALT-associated PML bodies. Mol. Cell 81 (5), 1027–1042.e4. 10.1016/j.molcel.2020.12.030 33453166 PMC8245000

[B169] Zheng J. Li Z. Min W. (2022). Current status and future promise of next-generation poly (ADP-Ribose) polymerase 1-selective inhibitor AZD5305. Front. Pharmacol. 13, 979873. 10.3389/fphar.2022.979873 36756144 PMC9899804

[B170] Zheng X. H. Nie X. Fang Y. Zhang Z. Xiao Y. Mao Z. (2017). A cisplatin derivative tetra-Pt(bpy) as an oncotherapeutic agent for targeting ALT cancer. J. Natl. Cancer Inst. 109 (10). 10.1093/jnci/djx061 28521363

[B171] Zimmermann A. Zenke F. T. Chiu L. Y. Dahmen H. Pehl U. Fuchss T. (2022). A new class of selective ATM inhibitors as combination partners of DNA double-strand break inducing cancer therapies. Mol. Cancer Ther. 21 (6), 859–870. 10.1158/1535-7163.MCT-21-0934 35405736 PMC9381122

